# Sitagliptin Modulates Functional and Phenotypic Properties of Human Neutrophils Under Normal- and High-Glucose Conditions In Vitro

**DOI:** 10.3390/molecules31081257

**Published:** 2026-04-10

**Authors:** Vanja Mališ, Marija Drakul, Sara Rakočević, Ljiljana Kozić, Anđela Dubovina, Darinka Popović, Dejan Bokonjić, Dušan Mihajlović, Miodrag Čolić

**Affiliations:** 1 Faculty of Medicine Foča, University of East Sarajevo, Studentska 5, 73300 Foča, Bosnia and Herzegovina; vanjamalis916@gmail.com (V.M.); marijadrakul@gmail.com (M.D.); saradrakocevic@gmail.com (S.R.); ljiljanakozic8@gmail.com (L.K.); mandicandjela21@gmail.com (A.D.); darinkadubovina91@gmail.com (D.P.); dbokonjic@gmail.com (D.B.); dusan.a.mihajlovic@gmail.com (D.M.); 2 Medical Faculty of the Military Medical Academy, University of Defense in Belgrade, Crnotravska 27, 11040 Belgrade, Serbia; 3 Serbian Academy of Sciences and Arts, Kneza Mihajla 35, 11000 Belgrade, Serbia

**Keywords:** sitagliptin, neutrophils, oxidative stress, neutrophil extracellular traps, surface markers

## Abstract

Sitagliptin is a dipeptidyl peptidase-4 (DPP-4) inhibitor used to treat type 2 diabetes. However, several studies have demonstrated its anti-inflammatory and immunomodulatory properties. The aim of this study was to investigate the effect of sitagliptin on the functional and phenotypic properties of human neutrophils under normal (NG, 5.5 mM)- and high (HG, 22 mM)-glucose conditions in vitro. Neutrophils were pretreated with varying concentrations of sitagliptin and stimulated with phorbol-12-myristate-13-acetate (PMA), N-formyl-L-methionyl-L-leucyl-L-phenylalanine (fMLP), calcium ionophore (CaI), or opsonized zymosan (OpZym). Survival, phenotypic, and functional characteristics were then assessed. Our results showed that sitagliptin was non-cytotoxic to neutrophils even at very high concentrations. It decreased the production of reactive oxygen species (ROS) and neutrophil extracellular traps (NETs), generally following a stimulus- and concentration-dependent pattern. The effect was more pronounced under HG conditions. Furthermore, sitagliptin showed a significant ROS-scavenging effect in a cell-free system. It also rapidly altered the expression of surface markers in both resting and fMLP-stimulated neutrophils, typically upregulating CD10, CD16, CD62L, CD63, CD88, CD89, and PD-L1, and downregulating CD11b/CD18, CD66b, and CD182, a phenotype consistent with a dampened, less-primed activation state of these cells. In conclusion, sitagliptin exhibited marked antioxidative/ROS-scavenging activity in neutrophil cultures and induced a coordinated shift in neutrophil phenotype, accompanied by suppression of NETosis under both NG and HG conditions. Collectively, these data support the view that neutrophils may constitute an additional cellular target contributing to sitagliptin’s anti-inflammatory and immunomodulatory profile.

## 1. Introduction

Selective DPP-4 inhibitors (DPP-4i), often known as gliptins, are a relatively new class of oral antidiabetic medications [1]. Sitagliptin was the first selective DPP-4i approved for the treatment of type 2 diabetes mellitus (T2DM) in 2006, followed by the development of several other selective DPP-4i (saxagliptin, vildagliptin, linagliptin, teneligliptin, and alogliptin) [2,3]. Chemically, sitagliptin is a fluorinated triazolopyrazine derivative. Pharmacologically, it inhibits both membrane-bound and soluble DDP-4, thereby preventing the degradation of incretin hormones. As a result, glucose-dependent insulin secretion increases, and glucagon secretion decreases. Sitagliptin is administered orally, has a low incidence of adverse events, including hypoglycemia, and is weight-neutral [2,3,4].

Given the widespread expression of DPP-4/CD26, a growing body of recent studies has found that DPP-4i may have additional benefits beyond their effects on glucose metabolism. Clinical studies and in vitro research suggest that this class of inhibitors may have immunomodulatory effects, which could be beneficial in the treatment of autoimmune and inflammatory disorders [5,6]. Several studies have demonstrated the role of DPP-4 and DPP-4i in adaptive immune responses, and an understanding of their importance in innate immunity is emerging. DPP-4 inhibitors have been shown to affect innate immunity, specifically vascular endothelial cells, neutrophils, and monocyte/macrophage responses, while DPP-4′s varied substrates play multiple functions in innate immunity, indicating that it may impact several pathways [7,8].

Neutrophils are highly specialized antimicrobial immune cells that respond initially to infections. To eliminate invading pathogens, neutrophils use cellular and noncellular mechanisms, including phagocytosis, the release of antimicrobial and protease molecules, and the production of reactive oxygen species. Several studies, mostly in vitro, have shown that DPP-4i can modify certain functional and phenotypic characteristics of neutrophil granulocytes [8].

Chronic hyperglycemia associated with diabetes primes neutrophils. These cells produce increased amounts of neutrophil extracellular traps (NETs) [9] and reactive oxygen species (ROS) [10], which drive inflammation and tissue damage. Abnormal or dysregulated neutrophil function has been reported as a cause of increased infection episodes among patients with diabetes. These changes in neutrophils include increased extracellular superoxide production [11], reduced neutrophil chemotaxis, and decreased bacterial-killing capacity [12]. The research found that prolonged exposure to elevated glucose levels led neutrophils to switch to a “nonactivated” state and become resistant to immunological stimulation. The authors propose that pathologic glucose levels trigger a brief neutrophil activation, followed by suppression of cell activity, which may lead to nonspecific tissue damage and increased vulnerability to infections [13].

In an in vitro human neutrophil model, the effects of five DPP-4i were studied. Linagliptin showed a dose-dependent (1 µM to 100 µM) effect on ROS production by LPS-stimulated neutrophils, whereas the other investigated DPP-4i (sitagliptin, vildagliptin, alogliptin, and saxagliptin) had no such effect [14]. Treatment of the lung allograft with the irreversible DPP-4i AB192 (bis(4-acetamidophenyl) 1-(s)-prolylpyrrolidine 2(r,s)-phosphonate) before surgical transplantation improved ultrastructure and reduced neutrophil infiltration of the allograft compared with untreated controls. The authors also showed that DPP-4i treatment was associated with increased expression of vasoactive intestinal polypeptide (VIP) in lung grafts [15]. VIP has previously been shown to reduce neutrophil infiltration, suggesting a possible indirect effect of DPP-4i on neutrophil activity [16]. An experimental study in a mouse model of dextran sulfate-induced colitis showed that DPP-4i administration decreased myeloperoxidase activity [16]. While the current study suggests that DPP-4i can influence certain aspects of neutrophil activity in experimental models of inflammation, the therapeutic implications of its use and the specific mechanisms of action remain unknown. Enzymatically active DPP-4/CD26 can act as a chemorepellent for neutrophils. Using an in vitro chemotaxis model, it was shown that both non-stimulated and TNF-α-stimulated neutrophils migrate dose-dependently from the site of recombinant DPP-4 application [17]. When the non-selective DPP-4i Diprotin A and DPP-1c hydrochloride are added in combination with a recombinant DPP-4 gradient, the chemorepulsive activity is significantly attenuated, suggesting that the ability of DPP-4 to act as a chemorepellent depends on its enzymatic activity [18]. The authors showed that treatment of mice with aerosolized DPP-4 reduced bleomycin-induced neutrophil infiltration, whereas no changes were observed in other inflammatory cell types [8]. Although evidence suggests that DPP-4i can affect neutrophil recruitment and activity, the exact mechanisms remain unknown. DPP-4i may have both direct and indirect effects on neutrophil activity in specific inflammatory conditions [6], but further research is needed to determine its effects and potential therapeutic applications. The aim of this study was to investigate the effects of non-toxic concentrations of sitagliptin on human peripheral blood neutrophils cultured in vitro under normal- and high-glucose conditions. Resting cells and cells activated by pharmacological agents or microbial stimuli were assessed for ROS production, NET formation, and phenotypic characteristics.

## 2. Results

### 2.1. Effects of Different Concentrations of Sitagliptin on Apoptosis of Human Peripheral Blood Neutrophils

Before functional experiments, the cytotoxic potential of sitagliptin toward human neutrophils was evaluated. Purified peripheral blood neutrophils were incubated for 16 h under normal-glucose (NG) or high-glucose (HG) conditions with sitagliptin (3.7–2000 µg/mL), in the presence or absence of N-formyl-methionyl-leucyl-phenylalanine (fMLP).

As shown in Table 1 and Supplementary Figure S1, unstimulated neutrophils exhibited high and comparable levels of spontaneous apoptosis under both NG and HG conditions, which increased further following fMLP stimulation. Treatment with sitagliptin at low-to-moderate concentrations (3.7–250 µg/mL) did not significantly affect apoptosis in either unstimulated or fMLP-stimulated neutrophils cultured under NG or HG conditions. In contrast, the highest tested concentration of sitagliptin (2000 µg/mL) was cytotoxic, resulting in a significant increase in the proportion of apoptotic cells both in NG (*p* < 0.05 and *p* < 0.01) and HG medium (*p* < 0.005). However, exposure to sitagliptin at higher non-cytotoxic concentrations (500 and 1000 µg/mL) was associated with a concentration-dependent reduction in neutrophil apoptosis (*p* < 0.05, *p* < 0.01, and *p* < 0.005). The reduction in apoptosis observed at 1000 µg/mL was more pronounced under HG conditions.

### 2.2. The Impact of Sitagliptin on the Respiratory Burst of Human Neutrophils

Neutrophils were cultured under either unstimulated or stimulated conditions using pharmacological agents or microbial products that differentially activate the oxidative burst. The culture medium contained either normal- or high-glucose concentrations, as described above. Total ROS were measured using luminol, whereas extracellular ROS were measured using isoluminol. Luminol-based chemiluminescence reflects total ROS production because luminol can diffuse across cell membranes, whereas isoluminol, due to its lower membrane permeability and extracellular horseradish peroxidase (HRP)-dependent oxidation, predominantly detects extracellular ROS.

#### 2.2.1. Effect of Sitagliptin on ROS Production in Unstimulated Neutrophil Cultures Under Normal- and High-Glucose Conditions

**Figure 1:**
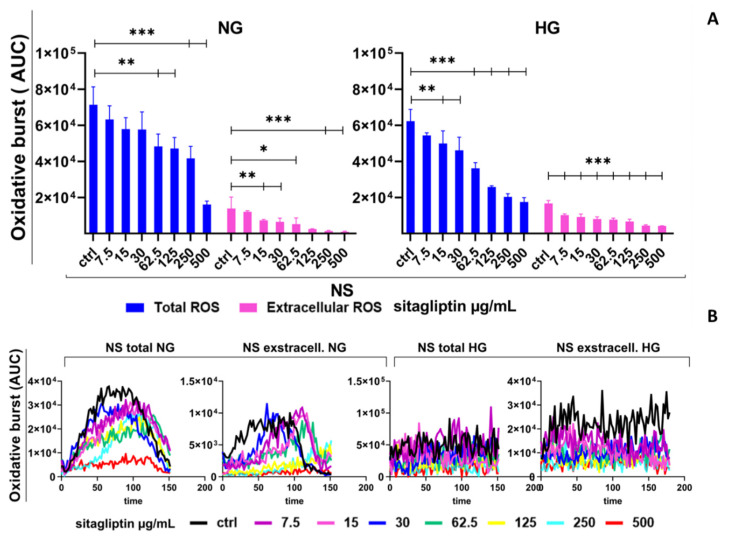
Effect of different concentrations of sitagliptin on the oxidative burst of human peripheral blood neutrophils. Neutrophils were cultured in HBSS+ medium containing either normal glucose (NG, 5.5 mM) or high glucose (HG, 22 mM) for 2 h. Cells were then treated with sitagliptin (7.5, 15, 30, 62.5, 125, 250, or 500 µg/mL) for 1 h. Cultures were non-stimulated (NS), and oxidative burst was measured using luminol (total ROS, 50 µM) and isoluminol (extracellular ROS, 50 µM). (**A**) Results are presented as mean ± SD (*n* = 3 independent donors). * *p* < 0.05, ** *p* < 0.01, *** *p* < 0.005, relative to the corresponding controls (neutrophils not treated with sitagliptin, Ctrl). (**B**) Values are expressed as the area under the curve (AUC) relative to the control group of neutrophils not treated with sitagliptin (Ctrl). Representative plots of the oxidative burst (AUC) from one experiment are shown.

Resting neutrophils produce relatively low levels of total ROS (approximately 7 × 10^4^ AUC values). Basal extracellular ROS levels were approximately fourfold lower than total ROS. Under NG conditions, sitagliptin significantly reduced total ROS production in unstimulated neutrophil cultures at concentrations of 62.5, 125, 250, and 500 µg/mL (*p* < 0.01 and *p* < 0.005). However, sitagliptin had a more pronounced inhibitory effect on extracellular ROS, with statistically significant inhibition observed at concentrations ranging from 15 to 500 µg/mL (*p* < 0.05, *p* < 0.01, and *p* < 0.005) (Figure 1).

Under HG conditions, basal total ROS levels were similar to those observed under NG conditions. Sitagliptin reduced total ROS levels in a concentration-dependent manner, more effectively than under NG conditions, starting at 15 µg/mL (*p* < 0.01 and *p* < 0.005). Extracellular ROS decreased across the entire tested concentration range (7.5–500 µg/mL) (*p* < 0.005). (Figure 1). Sitagliptin exerted an inhibitory effect on intracellular ROS, as shown by a concentration-dependent decrease in mean fluorescence intensity (MFI) in DHR-staining experiments (Supplementary Figure S2).

#### 2.2.2. Effect of Sitagliptin on Phorbol 12-Myristate 13-Acetate-Induced ROS Production Under Normal- and High-Glucose Conditions

**Figure 2:**
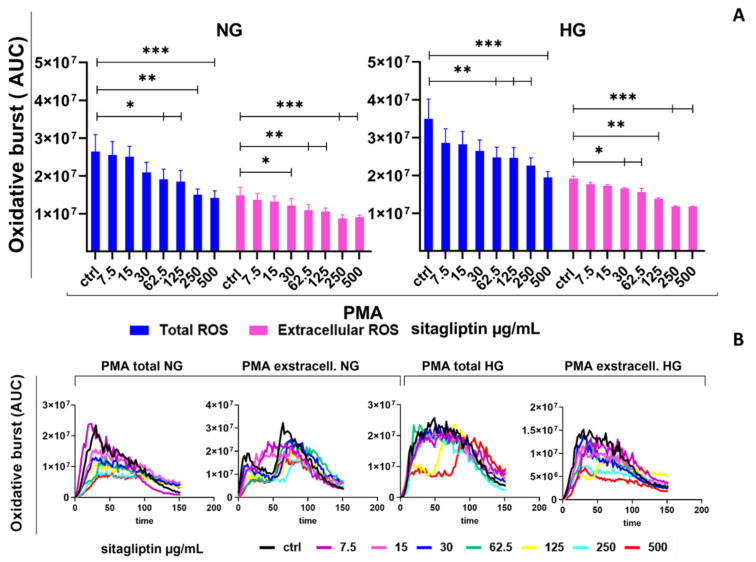
Effect of different concentrations of sitagliptin on the oxidative burst of phorbol 12-myristate 13-acetate (PMA)-stimulated human peripheral blood neutrophils. Neutrophils were cultured in HBSS+ medium containing either normal glucose (NG, 5.5 mM) or high glucose (HG, 22 mM) for 2 h. Cells were then treated with sitagliptin (7.5, 15, 30, 62.5, 125, 250, or 500 µg/mL) for 1 h. The cultures were stimulated with PMA (50 nM) as described in Materials and Methods, and the oxidative burst was measured using luminol (total ROS, 50 µM) and isoluminol (extracellular ROS, 50 µM). (**A**) Results are presented as mean ± SD (*n* = 3 independent donors). * *p* < 0.05, ** *p* < 0.01, *** *p* < 0.005, relative to the corresponding controls (neutrophils not treated with sitagliptin, Ctrl). (**B**) Values are expressed as the area under the curve (AUC) relative to the control group of neutrophils not treated with sitagliptin (Ctrl). Representative plots of the oxidative burst (AUC) from one experiment are shown.

Stimulation with phorbol 12-myristate 13-acetate (PMA) triggered a strong, prolonged (more than 150 min) oxidative burst in human neutrophils. Based on AUC data, PMA increased total ROS from approximately 7 × 10^4^ under basal conditions (Figure 1) to approximately 2.5 × 10^7^ under PMA stimulation (Figure 2), representing a roughly 350-fold increase. Extracellular ROS accounted for about 55–60% of the total oxidative burden (approximately 1.5 × 10^7^ AUC units). Sitagliptin decreased PMA-induced total ROS levels in a concentration-dependent manner. Statistically significant inhibition began at mid-range concentrations (62.5 µg/mL) (*p* < 0.05) and reached maximum suppression at 500 µg/mL (*p* < 0.005). This resulted in a 50% reduction in ROS compared with the PMA control.

Extracellular ROS were also decreased, with reductions starting at 30 µg/mL (*p* < 0.05). As with total ROS, inhibition of the oxidative burden was more pronounced at higher concentrations, resulting in a roughly 50% decrease at 500 µg/mL (*p* < 0.005) (Figure 2).

HG conditions further intensified the PMA-driven response, as evidenced by higher total ROS (approximately 3.5 × 10^7^ AUC units) and extracellular ROS (approximately 1.8 × 10^7^ AUC units) than in NG, corresponding to approximately 50% of the total ROS levels (Figure 2). In HG cultures, sitagliptin reduced total ROS to a similar extent as under NG conditions, with the greatest inhibition at 500 µg/mL (approximately a 40% decrease relative to the HG PMA control) (*p* < 0.005). Sitagliptin showed a similar inhibition pattern of extracellular ROS to that observed under NG conditions (*p* < 0.05, *p* < 0.01, and *p* < 0.005) (Figure 2).

#### 2.2.3. Effect of Sitagliptin on fMLP-Induced ROS Production Under Normal- and High-Glucose Conditions

**Figure 3:**
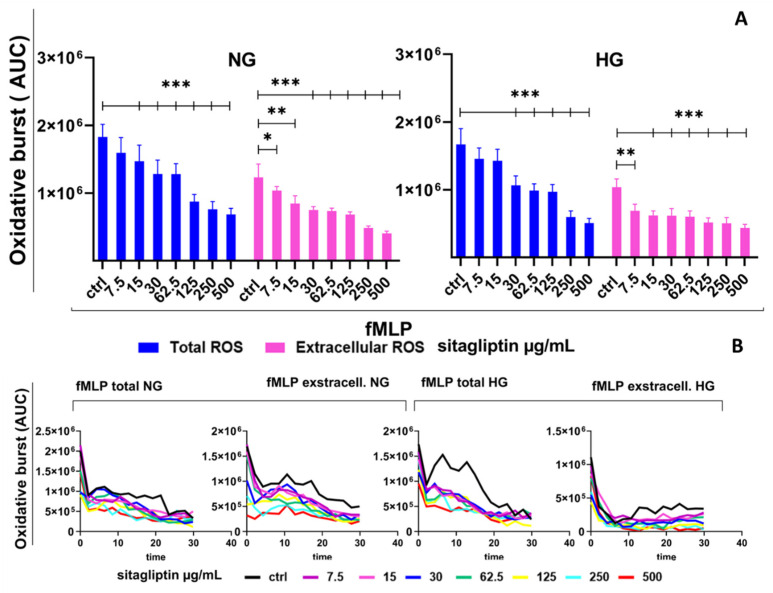
Effect of varying concentrations of sitagliptin on the oxidative burst of N-formyl-methionyl-leucyl-phenylalanine (fMLP)-stimulated human peripheral blood neutrophils. Neutrophils were cultured in HBSS+ medium containing either normal glucose (NG, 5.5 mM) or high glucose (HG, 22 mM) for 2 h. Cells were then treated with sitagliptin (7.5, 15, 30, 62.5, 125, 250, or 500 µg/mL) for 1 h. The cultures were stimulated with fMLP (1 µM) as described in Materials and Methods, and the oxidative burst was measured using luminol (total ROS, 50 µM) and isoluminol (extracellular ROS, 50 µM). (**A**) Results are presented as mean ± SD (*n* = 3 independent donors). * *p* < 0.05, ** *p* < 0.01, *** *p* < 0.005, relative to the corresponding controls (neutrophils not treated with sitagliptin, Ctrl). (**B**) Values are expressed as the area under the curve (AUC) relative to the control group of neutrophils not treated with sitagliptin (Ctrl). Representative plots of the oxidative burst (AUC) from one experiment are shown.

Stimulation of human neutrophils with fMLP induced a pronounced but relatively short (30 min) oxidative burst compared with unstimulated cultures. Based on AUC values, fMLP increased total ROS to approximately 1.8 × 10^6^ AUC units (Figure 3), corresponding to a 25-fold increase. Extracellular ROS accounted for a substantial fraction of the response (approximately 1.2 × 10^6^ AUC units), about 2/3 of the total ROS. Sitagliptin reduced fMLP-induced total ROS in a concentration-dependent manner (*p* < 0.005), starting at a low concentration (15 µg/mL). The greatest suppression was observed at 500 µg/mL (approximately a 65% reduction relative to the fMLP control). Extracellular ROS were likewise reduced dose-dependently. The inhibition was statistically significant even at the lowest concentration (7.5 µg/mL) (*p* < 0.05) and reached a similar 65% decrease in ROS levels at 500 µg/mL (*p* < 0.005) (Figure 3).

Under HG conditions, the fMLP-induced oxidative burst was generally similar in magnitude to that observed under NG conditions for both total and extracellular ROS (Figure 3). Sitagliptin consistently decreased both total and extracellular ROS in a dose-dependent way, with a stronger effect on extracellular ROS. The greatest inhibition occurred at higher concentrations, reaching maximum suppression at 500 µg/mL (*p* < 0.005). The maximum reduction for both total and extracellular ROS levels was around 60% (*p* < 0.005) (Figure 3). Sitagliptin also inhibited intracellular ROS in fMLP-stimulated neutrophil cultures, as shown by a concentration-dependent decrease in MFI in DHR-staining experiments (Supplementary Figure S2).

#### 2.2.4. Effect of Sitagliptin on Calcium Ionophore-Induced ROS Production Under Normal- and High-Glucose Conditions

**Figure 4:**
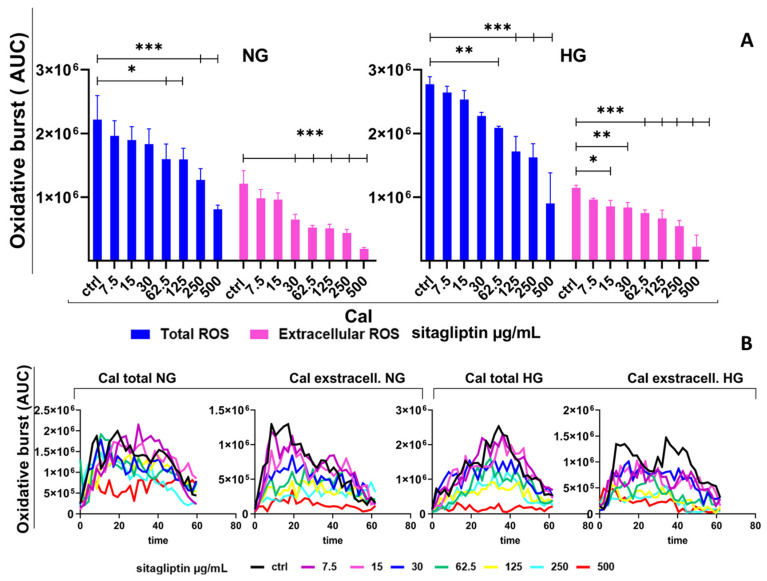
Effect of different concentrations of sitagliptin on the oxidative burst of calcium ionophore (CaI)-stimulated human peripheral blood neutrophils. Neutrophils were cultured in HBSS+ medium containing either normal glucose (NG, 5.5 mM) or high glucose (HG, 22 mM) for 2 h. Cells were then treated with sitagliptin (7.5, 15, 30, 62.5, 125, 250, or 500 µg/mL) for 1 h. The cultures were stimulated with CaI (1 µM) as described in Materials and Methods, and the oxidative burst was measured using luminol (total ROS, 50 µM) and isoluminol (extracellular ROS, 50 µM). (**A**) Results are presented as mean ± SD (*n* = 3 independent donors). * *p* < 0.05, ** *p* < 0.01, *** *p* < 0.005, relative to the corresponding controls (neutrophils not treated with sitagliptin, Ctrl). (**B**) Values are expressed as the area under the curve (AUC) relative to the control group of neutrophils not treated with sitagliptin (Ctrl). Representative plots of the oxidative burst (AUC) from one experiment are shown.

Stimulation of human neutrophils with the calcium ionophore (CaI) induced a pronounced oxidative burst compared with unstimulated cultures. Based on AUC values, CaI increased total ROS to approximately 2.2 × 10^6^ AUC units (Figure 4), corresponding to a 27-fold increase. The ROS response usually terminated for about 60 min. Extracellular ROS accounted for a substantial fraction of the response (approximately 1.2 × 10^6^ AUC units). Sitagliptin reduced CaI-induced total ROS levels in a concentration-dependent manner, with statistically significant differences observed at concentrations of 62.5 µg/mL or higher (*p* < 0.05 and *p* < 0.005). The reduction at the highest concentration was about 65% relative to the CaI control. Extracellular ROS levels were further attenuated in a concentration-dependent manner, starting at 15 µg/mL and reaching an 85% decrease at 500 µg/mL (*p* < 0.005) (Figure 4).

Under HG conditions, CaI-induced total ROS was higher than under NG, reaching approximately 2.8–2.9 × 10^6^ AUC units. However, CaI-induced extracellular ROS was broadly comparable to NG at baseline stimulation (Figure 4). Sitagliptin again suppressed both readouts in a dose-dependent manner, as in NG cultures, with maximal inhibition at 500 µg/mL (approximately 65–70% reduction in total ROS; *p* < 0.005) and about 75–80% reduction in extracellular ROS (*p* < 0.005; Figure 4).

#### 2.2.5. Effect of Sitagliptin on Opsonized Zymosan-Induced ROS Production Under Normal- and High-Glucose Conditions

**Figure 5:**
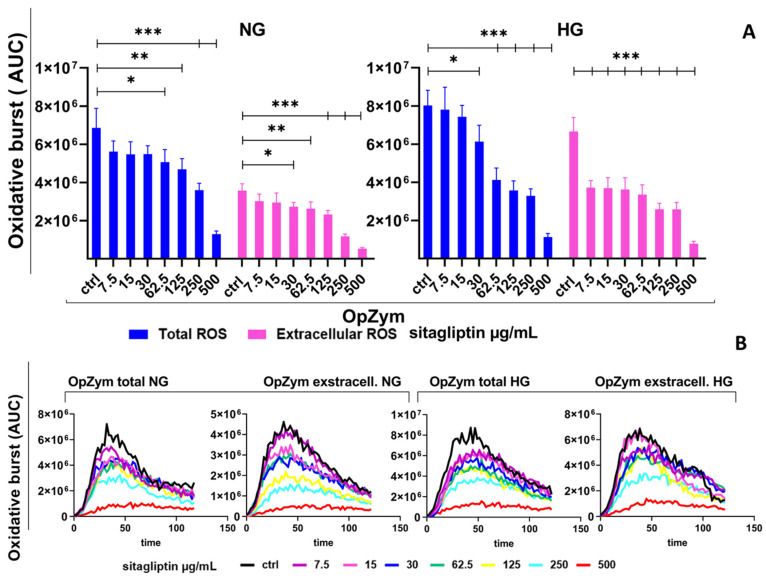
Effect of different concentrations of sitagliptin on the oxidative burst of opsonized zymosan (OpsZym)-stimulated human peripheral blood neutrophils. Neutrophils were cultured in HBSS+ medium containing either normal glucose (NG, 5.5 mM) or high glucose (HG, 22 mM) for 2 h. Cells were then treated with sitagliptin (7.5, 15, 30, 62.5, 125, 250, or 500 µg/mL) for 1 h. The cultures were stimulated with OpsZym (10 µg/mL) as described in Materials and Methods, and the oxidative burst was measured using luminol (total ROS, 50 µM) and isoluminol (extracellular ROS, 50 µM). (**A**) Results are presented as mean ± SD (*n* = 3 independent donors). * *p* < 0.05, ** *p* < 0.01, *** *p* < 0.001, relative to the corresponding controls (neutrophils not treated with sitagliptin, Ctrl). (**B**) Values are expressed as the area under the curve (AUC) relative to the control group of neutrophils not treated with sitagliptin (Ctrl). Representative plots of the oxidative burst (AUC) from one experiment are shown.

Stimulation of human neutrophils with opsonized zymosan (OpsZym) induced a strong, prolonged oxidative burst (about 125 min) compared with unstimulated cultures. Based on AUC values, OpsZym increased total ROS to 7 × 10^6^ AUC units (Figure 5), representing a 100-fold increase. Extracellular ROS accounted for a substantial fraction of the response (approximately 4.0 × 10^6^ AUC units).

Sitagliptin reduced OpsZym-induced total ROS in a concentration-dependent manner, showing statistically significant inhibition at mid-range concentrations (62.5 µg/mL) (*p* < 0.05) and the greatest suppression at 500 µg/mL (approximately 85% reduction vs. OpsZym control) (*p* < 0.005). Extracellular ROS were also decreased, with reductions starting at 30 µg/mL (*p* < 0.05). As with total ROS, inhibition of the oxidative burden was more pronounced at higher concentrations, resulting in about 90% suppression at 500 µg/mL (*p* < 0.005) (Figure 5).

Under HG conditions, the neutrophil oxidative burst was further potentiated by OpsZym, as evidenced by higher total ROS levels (approximately 8 × 10^6^ AUC units). Notably, HG conditions produced a particularly strong increase in extracellular ROS (approximately 7 × 10^6^ AUC units), comparable to total ROS levels in NG neutrophil cultures. In HG cultures, sitagliptin again suppressed both total (*p* < 0.05 and *p* < 0.005) and, particularly, extracellular ROS, following a pattern similar to that observed under NG conditions (*p* < 0.005) (Figure 5).

### 2.3. Effect of Sitagliptin on Scavenging Activity

#### 2.3.1. Hydrogen Peroxide Scavenging Activity

**Figure 6:**
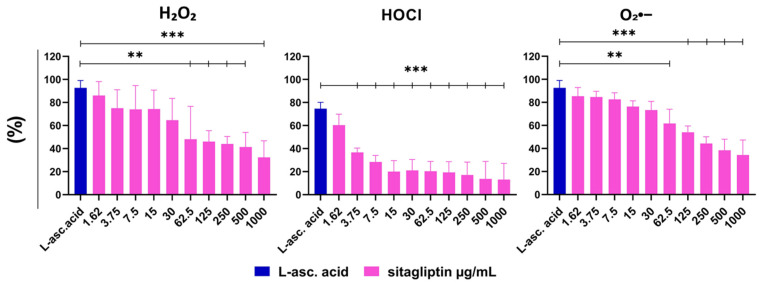
Effect of varying concentrations of sitagliptin on scavenging of H_2_O_2_, HOCl, and superoxide (O_2_•^−^). Scavenging activity was assessed in three complementary cell-free systems in the presence of sitagliptin at concentrations of 1.62, 2.75, 3.75, 7.5, 15, 30, 62.5, 125, 250, 500, and 1000 µg/mL, as described in Section 4. H_2_O_2_ scavenging was quantified by measuring the inhibition of luminol oxidation (chemiluminescence 50 µM), HOCl scavenging by reduced conversion of dihydrorhodamine (DHR 5 µM) to rhodamine 123 (fluorescence), and superoxide scavenging by inhibition of NBT (43 µM) reduction to formazan (spectrophotometry). L-ascorbic acid (500 µM) served as a positive control. Data represent four independent experiments, each performed in triplicate, and are shown as mean ± SD (*n* = 4). ** *p* < 0.01, *** *p* < 0.005 versus L-ascorbic acid.

Using a previously described chemiluminescence assay, we first evaluated H_2_O_2_-scavenging activity to assess sitagliptin’s antioxidant potential. L-ascorbic acid, a well-known dietary anti-oxidant, served as the positive control. Sitagliptin was tested across a dose range of 1.62 to 1000 µg/mL. Compared with the positive control, sitagliptin exhibited dose-dependent scavenging activity, with statistically significant effects at 62.5–1000 µg/mL. Notably, sitagliptin at 250 and 500 µg/mL achieved approximately 43% and 51% scavenging activity, respectively (*p* < 0.005) (Figure 6).

#### 2.3.2. Hypochlorous Acid Scavenging Activity

Hypochlorous acid (HOCl) scavenging was evaluated using a fluorescence-based microplate assay in which HOCl oxidizes dihydrorhodamine 123 (DHR) to rhodamine 123. Sitagliptin demonstrated very efficient HOCl scavenging activity, compared with L-ascorbic acid (*p* < 0.005). At low concentrations (3.75–15 µg/mL), the effect was concentration-dependent and reached a plateau between 15 and 1000 µg/mL. At 500 µg/mL, sitagliptin exhibited 87% scavenging activity relative to the positive control, indicating particularly strong reactivity toward HOCl (Figure 6).

#### 2.3.3. Superoxide Radical Scavenging Activity

Superoxide (O_2_•^−^) scavenging was evaluated using the Nicotinamide Adenine Dinucleotide (NADH)/phenazine methosulfate (PMS) system to generate O_2_•^−^, followed by spectrophotometric quantification in a microplate format. Sitagliptin effectively scavenged superoxide radicals across the 62.5–1000 µg/mL range, relative to the positive control. At 250 and 500 µg/mL, sitagliptin exhibited 38% and 47% scavenging activity relative to the positive control (*p* < 0.01 and *p* < 0.005), confirming its moderate but meaningful reactivity toward superoxide (Figure 6).

### 2.4. Impact of Sitagliptin on Neutrophil Extracellular Trap Formation

**Figure 7:**
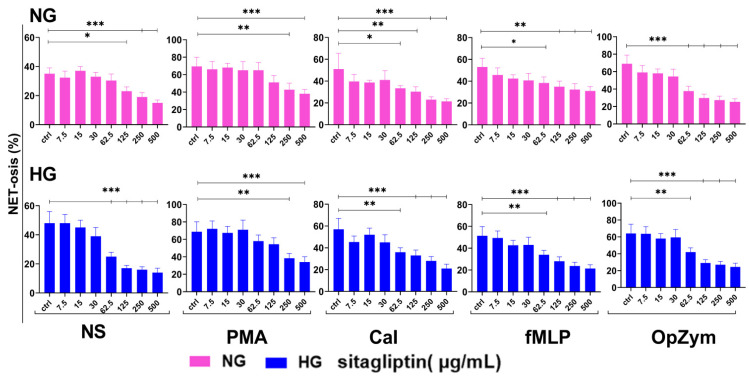
Neutrophils were cultured in HBSS^+^ medium containing either normal glucose (NG, 5.5 mM) or high glucose (HG, 22 mM) for 2 h, followed by treatment with sitagliptin (7.5, 15, 30, 62.5, 125, 250, and 500 µg/mL) as described in Materials and Methods. Cells were then left non-stimulated (NS) or stimulated with PMA (50 nM), CaI (1 µM), fMLP (1 µM), or OpZym (10 µg/mL) and incubated for an additional 4 h at 37 °C. NET formation was quantified by fluorescence measurement and is presented as the percentage of NETosis relative to the total DNA extracted after Triton X-100 treatment (1%). Data represent mean ± SD (*n* = 3 independent donors). * *p* < 0.05, ** *p* < 0.01, *** *p* < 0.005 vs. corresponding untreated control neutrophils (Ctrl). Abbreviations: NG—normal glucose; HG—high glucose; NS—non-stimulated; PMA—phorbol 12-myristate 13-acetate; CaI—calcium ionophore A23187; fMLP—N-formylmethionyl-leucyl-phenylalanine; OpZym—opsonized zymosan; NETs—neutrophil extracellular traps; Ctrl—control.

Basal neutrophil extracellular trap (NET) formation in unstimulated NG conditions accounted for about 35% of total DNA release in the Triton-X-positive control. Sitagliptin reduced NET formation under these culture conditions in a dose-dependent manner at higher concentrations (125–500 µg/mL) (*p* < 0.05 and *p* < 0.005) (Figure 7). Baseline NETosis was higher under HG (about 20%) than basal NETosis in NG cultures. Sitagliptin significantly reduced spontaneous NET formation at 62.5–500 µg/mL under HG conditions (*p* < 0.005) (Figure 7).

NETosis was about two-fold higher following PMA stimulation under both glucose conditions than in NS neutrophil cultures. Sitagliptin markedly inhibited NET formation in both NG and HG cultures (70–80% inhibition), but only at the highest concentrations (250 and 500 µg/mL) (*p* < 0.01 and *p* < 0.005, respectively) (Figure 7).

CaI also stimulated NETosis independently of glucose concentrations in the culture medium. Sitagliptin also reduced NETosis in a dose-dependent manner from 62.5 to 500 µg/mL. Inhibition at the highest sitagliptin concentration was about 60% (NG) (*p* < 0.05, *p* < 0.01, and *p* < 0.005) and 70% (HG) (*p* < 0.01 and *p* < 0.005), respectively (Figure 7).

Similarly, fMLP stimulation induced moderate NETosis, which was significantly reduced by sitagliptin across 62.5–500 µg/mL in both glucose environments. The inhibitory effect increased with increasing sitagliptin concentrations, reaching about 40% (NG) (*p* < 0.01) and 55% (HG) at 500 µg/mL (*p* < 0.005) (Figure 7).

OpZym induced a strong NETotic response, comparable to that of PMA. Sitagliptin significantly reduced OpZym-induced NET formation at 62.5–500 µg/mL under both glucose concentrations. Inhibition at the highest sitagliptin concentration was 70–75% (*p* < 0.005) (Figure 7).

Overall, moderate-to-high concentrations of sitagliptin consistently suppressed NET formation across all stimuli and glucose conditions. The inhibitory effect was more pronounced under HG conditions and generally mirrored the pattern of ROS levels.

### 2.5. Effect of Sitagliptin on Phenotypic Properties of Resting and Activated Neutrophils

The next part of our study examined the effect of sitagliptin on the phenotypic properties of unstimulated and fMLP-stimulated neutrophils. Neutrophils were cultured under NG and HG conditions, as described in Materials and Methods. Twelve markers were analyzed after a short-term (30 min) fMLP stimulation.

#### 2.5.1. Effect of Sitagliptin on Markers Involved in Neutrophil Recruitment and Adhesion

**Figure 8:**
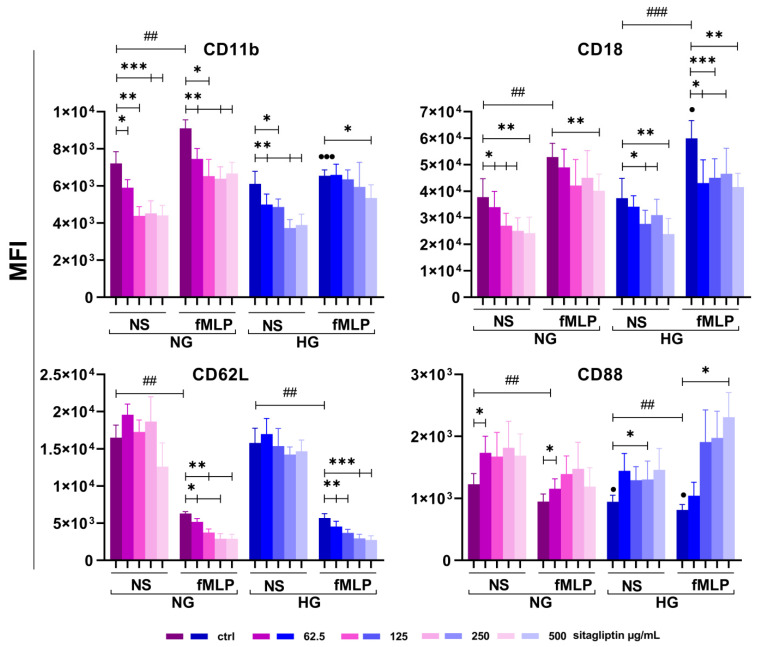
Effect of sitagliptin on the expression of CD11b, CD18, CD62L, and CD88. Human neutrophils were cultured in HBSS^+^ medium under normal-glucose (NG, 5.5 mM) or high-glucose (HG, 22 mM) conditions for 2 h, then treated with sitagliptin (62.5, 125, 250, and 500 µg/mL) for 60 min. Cells were left unstimulated (NS) or stimulated with fMLP (1µM). Surface expression of the markers was analyzed by flow cytometry and expressed as mean fluorescence intensity (MFI). Data are presented as mean ± standard deviation (SD) from four independent donors. Pairwise comparisons were performed using a paired Student’s *t*-test, whereas multiple-group comparisons were conducted using repeated-measures one-way analysis of variance (ANOVA), followed by Dunnett’s post hoc test. Asterisks indicate effects of sitagliptin relative to the corresponding unstimulated or fMLP-stimulated control within the same glycemic condition (NG or HG) (* *p* < 0.05, ** *p* < 0.01, *** *p* < 0.005). Hash symbols indicate differences between unstimulated and fMLP-stimulated controls within the NG or HG conditions (## *p* < 0.01, ### *p* < 0.005). Black circles indicate differences between NG and HG conditions, either between unstimulated controls or between fMLP-stimulated controls (● *p* < 0.05, ●●● *p* < 0.005).

Four markers (CD62L, CD11b, CD18, and CD88) involved in neutrophil adhesion and recruitment were analyzed. The results are shown in Figure 8 and Supplementary Figure S3.

Almost all non-stimulated and fMLP-stimulated neutrophils cultured under both NG and HG conditions expressed CD11b. By mean fluorescence intensity (MFI), CD11b expression was lower in unstimulated neutrophil cultures under HG conditions than under NG conditions (*p* < 0.005). fMLP augmented CD11b expression on neutrophils in both groups, but the effect was statistically significant only in the NG medium culture (*p* < 0.01). Sitagliptin decreased CD11b expression on unstimulated neutrophils under both glucose conditions across all concentrations tested (*p* < 0.05, *p* < 0.01, and *p* < 0.005). A similar down-modulatory effect of sitagliptin was observed in fMLP-stimulated cultures under NG conditions (*p* < 0.05 and *p* < 0.01). However, such an effect in fMLP-stimulated neutrophil cultures under HG conditions was observed only at the highest sitagliptin concentration (500 µg/mL) (*p* < 0.05).

CD18 expression on fMLP-activated neutrophils followed the same up-regulated pattern as CD11b. However, up-regulation was greater under HG conditions (*p* < 0.05). Sitagliptin inhibited CD18 expression on unstimulated neutrophils under both culture conditions in a concentration-dependent manner (*p* < 0.05 and *p* < 0.01). In fMLP-stimulated neutrophils cultured under NG conditions, sitagliptin significantly reduced CD18 expression only at the highest applied concentration (*p* < 0.01). In contrast, under HG conditions, a significant reduction in CD18 expression was observed at all tested concentrations (*p* < 0.05, *p* < 0.01, and *p* < 0.001).

Over 90% of neutrophils expressed CD62L. There were no statistically significant differences in the percentage or MFI of CD62L expression between NG and HG conditions. Stimulation of neutrophils with fMLP significantly reduced CD62L expression under both NG (*p* < 0.005) and HG (*p* < 0.005) culture conditions. Sitagliptin did not significantly alter CD62L expression in unstimulated neutrophils under either culture condition, but further reduced CD62L expression after fMLP stimulation, regardless of glucose levels, in a concentration-dependent manner (*p* < 0.05, *p* < 0.01, and *p* < 0.001).

CD88 was expressed on 25–35% of resting neutrophils, and both the percentage and MFI expression were higher under NG conditions (*p* < 0.05). fMLP decreased CD88 expression regardless of glucose concentrations (*p* < 0.01). Sitagliptin at 62.5 µg/mL (NG conditions) and 250 µg/mL (HG conditions) increased CD88 expression on resting neutrophils (*p* < 0.05). Sitagliptin augmented CD88 expression on fMLP-stimulated neutrophils in NG cultures at the lowest concentration (*p* < 0.05). This upregulating effect was observed in HG cultures at the highest sitagliptin concentration (*p* < 0.05).

#### 2.5.2. Effect of Sitagliptin on Neutrophil Chemotactic and Opsonin-Recognition Markers

**Figure 9:**
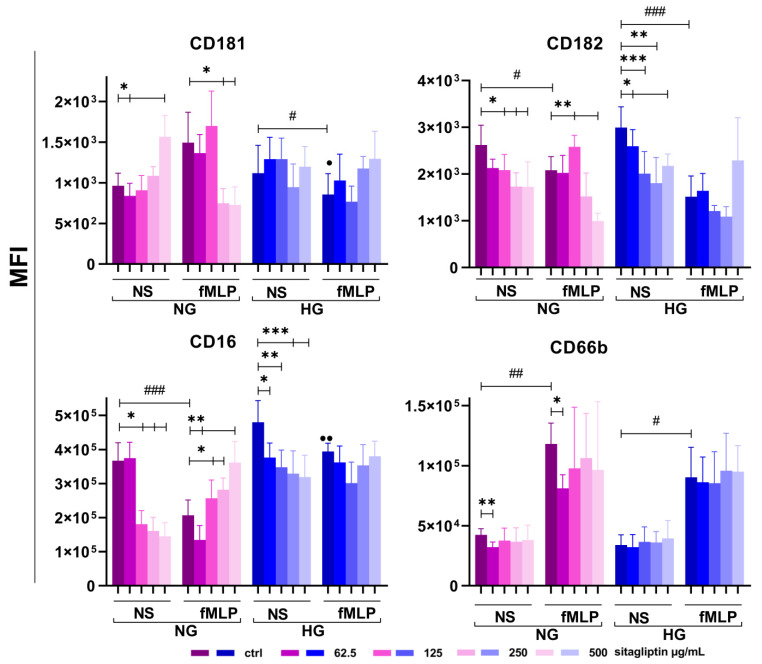
Effect of sitagliptin on the expression of CD181, CD182, CD16, and CD66b. Human neutrophils were cultured in HBSS^+^ medium under normal-glucose (NG, 5.5 mM) or high-glucose (HG, 22 mM) conditions for 2 h, followed by treatment with sitagliptin (62.5, 125, 250, and 500 µg/mL) for 60 min. Cells were left unstimulated (NS) or stimulated with fMLP. Surface expression of CD181, CD182, CD16, and CD66b was analyzed by flow cytometry and expressed as mean fluorescence intensity (MFI). Data are presented as mean ± standard deviation (SD) from four independent donors. Pairwise comparisons were performed using a paired Student’s *t*-test, whereas multiple-group comparisons were conducted using repeated-measures one-way analysis of variance (ANOVA), followed by Dunnett’s post hoc test. Asterisks indicate effects of sitagliptin compared with the corresponding unstimulated or fMLP-stimulated control within the same glycemic condition (NG or HG) (* *p* < 0.05, ** *p* < 0.01, *** *p* < 0.005). Hash symbols indicate differences between unstimulated and fMLP-stimulated controls within NG or HG conditions (# *p* < 0.05, ## *p* < 0.01, ### *p* < 0.005). Black circles indicate differences between NG and HG conditions, either between unstimulated controls or between fMLP-stimulated controls (● *p* < 0.05, ●● *p* < 0.01).

Four markers (CD181, CD182, CD16, and CD66b) involved in neutrophil chemotaxis, opsonization, and degranulation readout were analyzed. The results are shown in Figure 9 and Supplementary Figure S4.

About 20% of neutrophils expressed CD181. No statistically significant differences were observed in the percentage of CD181-positive cells or in CD181 MFI between NG and HG conditions. fMLP stimulation did not significantly modify CD181 MFI in both NG and HG neutrophil cultures. However, CD181 expression on fMPL-stimulated neutrophils was significantly higher in NG compared to HG conditions (*p* < 0.05). At the lowest concentration, sitagliptin significantly reduced CD181 expression on unstimulated neutrophils in NG medium (*p* < 0.05), whereas the highest concentration significantly increased its expression (*p* < 0.05). In the NG medium, sitagliptin inhibited CD181 expression in fMLP-stimulated neutrophils at higher concentrations (250 and 500 µg/mL) (*p* < 0.05), whereas it showed no modulatory effect under the corresponding HG conditions.

Approximately 75% of neutrophils expressed CD182 in both NG and HG cultures. fMLP stimulation significantly decreased CD182 MFI in NG (*p* < 0.05) and even more strongly in HG (*p* < 0.001). In unstimulated cultures, sitagliptin reduced CD182 expression, with a greater effect in HG (*p* < 0.05, *p* < 0.01, *p* < 0.005) than in NG (*p* < 0.05). In fMLP-stimulated NG cultures, sitagliptin showed a biphasic effect: up-regulation at 125 µg/mL; downregulation at 500 µg/mL (both *p* < 0.05). However, no significant changes were detected in corresponding HG cultures.

Almost all neutrophils (>95%) expressed CD16, with no significant difference in MFI between NG and HG conditions. fMLP stimulation significantly downregulated CD16 under NG conditions (*p* < 0.005) but not in HG medium, and the differences between these two groups were statistically significant (*p* < 0.01). In unstimulated neutrophils cultured in NG, sitagliptin reduced CD16 expression at 125–500 µg/mL (*p* < 0.05). The opposite effect was observed in fMLP-stimulated cultures at all sitagliptin concentrations (*p* < 0.05 and *p* < 0.01). Sitagliptin also reduced CD16 expression in HG cultures in a concentration-dependent manner (*p* < 0.05, *p* < 0.01, and *p* < 0.005). However, no effect was observed in fMLP-treated cultures at elevated glucose levels.

Almost all neutrophils (>98%) expressed CD66b in both media, with no statistically significant difference. fMLP stimulation significantly increased CD66b expression under NG (*p* < 0.01) and HG (*p* < 0.05) culture conditions. Sitagliptin, at the lowest concentration (62.5 µg/mL), reduced CD66b expression in unstimulated and fMLP-stimulated neutrophil NG cultures (*p* < 0.01 and *p* < 0.05, respectively). In contrast, sitagliptin did not significantly affect CD66b surface expression in resting or fMLP-stimulated cells under HG.

#### 2.5.3. Effect of Sitagliptin on Neutrophil Effector Readiness and Regulatory Phenotype Markers

**Figure 10:**
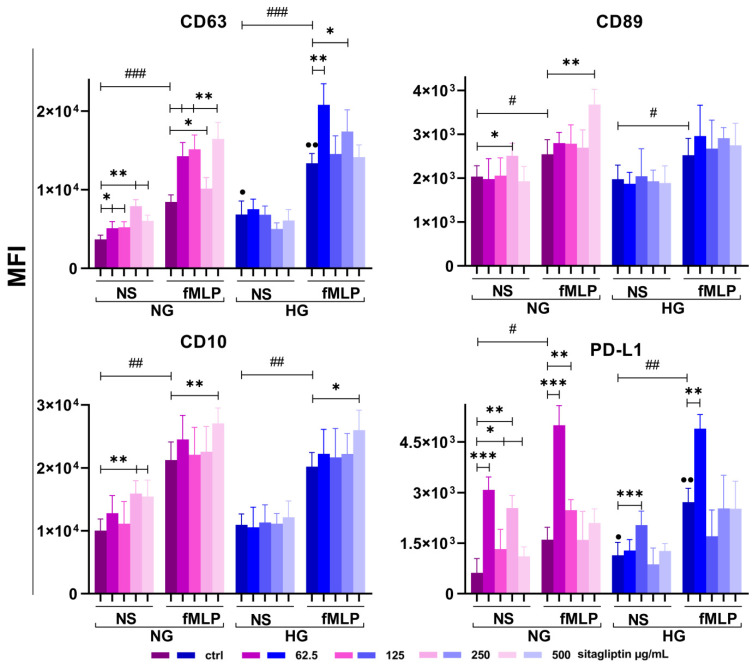
Effect of sitagliptin on CD63, CD89, CD10, and PD-L1 expression. Human neutrophils were cultured in HBSS^+^ medium under normal-glucose (NG, 5.5 mM) or high-glucose (HG, 22 mM) conditions for 2 h, then treated with sitagliptin (62.5, 125, 250, and 500 µg/mL) for 60 min. Cells were left unstimulated (NS) or stimulated with fMLP. Surface expression of CD63, CD89, CD10, and PD-L1 was analyzed by flow cytometry and expressed as mean fluorescence intensity (MFI). Data are presented as mean ± standard deviation (SD) from four independent donors. Pairwise comparisons were performed using a paired Student’s *t*-test, whereas multiple-group comparisons were conducted using repeated-measures one-way analysis of variance (ANOVA), followed by Dunnett’s post hoc test. Asterisks indicate effects of sitagliptin relative to the corresponding unstimulated or fMLP-stimulated control within the same glycemic condition (NG or HG) (* *p* < 0.05, ** *p* < 0.01, *** *p* < 0.005). Hash symbols indicate differences between unstimulated and fMLP-stimulated controls within the NG or HG conditions (# *p* < 0.05, ## *p* < 0.01, ### *p* < 0.005). Black circles indicate differences between NG and HG conditions, either between unstimulated controls or between fMLP-stimulated controls (● *p* < 0.05, ●● *p* < 0.01).

Four markers (CD63, CD89, CD10, and CD274) involved in neutrophil effector readiness and regulatory phenotype were analyzed. The results are shown in Figure 10 and Supplementary Figure S5.

CD63 was expressed on 55–70% resting neutrophils, and MFI increased with fMLP stimulation under both NG and HG conditions (*p* < 0.005). Expression was greater under HG conditions, both on unstimulated (*p* < 0.05) and fMLP-stimulated neutrophils (*p* < 0.01), compared to NG cultures. Sitagliptin, at all concentrations, up-regulated CD63 expression on unstimulated and fMLP-stimulated neutrophils under NG conditions (*p* < 0.05 and *p* < 0.01). However, the effect of sitagliptin was not observed in unstimulated cultures under HG conditions. Sitagliptin stimulated CD63 expression in fMLP-treated cultures under HG conditions, with statistically significant differences at 62.5 µg/mL (*p* < 0.01) and 250 µg/mL (*p* < 0.05).

CD89 was expressed on most (>90%) resting neutrophils, and MFI increased with fMLP stimulation under both glucose concentrations (*p* < 0.05). At higher concentrations (250 µg/mL) in unstimulated and 500 µg/mL (fMLP-stimulated) NG cultures, sitagliptin increased CD89 expression (*p* < 0.05 and *p* < 0.01, respectively). In contrast, sitagliptin did not significantly change CD89 expression under HG conditions.

CD10 was expressed on most (>90%) resting neutrophils, and MFI increased with fMLP stimulation under both NG and HG conditions (*p* < 0.005 and *p* < 0.01, respectively). Sitagliptin, at concentrations of 250 and 500 µg/mL, up-regulated CD10 expression on unstimulated neutrophils under NG conditions (*p* < 0.05); however, it was ineffective under HG conditions. Only the highest sitagliptin concentration up-regulated CD10 expression in fMLP-stimulated neutrophils, regardless of glucose concentration (*p* < 0.05).

CD274 (PD-L1) was expressed on 3–10% resting neutrophils, and MFI increased with fMLP stimulation under both NG and HG conditions (*p* < 0.05). The stimulatory effect of fMLP was more pronounced under HG conditions (*p* < 0.05). Sitagliptin, at concentrations of 62.5 and 250 µg/mL, up-regulated PD-L1 expression on unstimulated neutrophils under NG conditions (*p* < 0.05); however, it was ineffective under HG conditions. Only the lowest sitagliptin concentration (62.5 µg/mL) up-regulated PD-L1 expression in fMLP-stimulated neutrophils, independently of glucose concentrations (*p* < 0.05).

## 3. Discussion

This pioneering study examined how sitagliptin, a glucose-lowering drug, modulates key functional and phenotypic properties of human peripheral blood neutrophils. The choice of neutrophils as the experimental model is important because they are the major leukocyte subset in peripheral blood and key effector cells of innate immunity in infection and inflammation [19]. Moreover, accumulating evidence suggests that neutrophils play a significant role in the pathogenesis of diabetes-associated complications [20,21]. Accordingly, we cultured neutrophils under normal-glucose and hyperglycemic conditions in vitro to model diabetes-associated innate immune dysfunction.

Before the main experiments, we tested sitagliptin cytotoxicity to determine the optimal modulatory concentrations. We showed that human neutrophils are resistant to sitagliptin cytotoxicity, with only very high concentrations (2000 µg/mL) inducing apoptosis. These results are generally consistent with published data showing cytotoxic effects of this drug at 1000 µg/mL on human dendritic cells [22] and human peripheral blood lymphocytes [23], and about 500 µg/mL (1000 µM) on 3T3 fibroblasts [24]. The cytotoxic effect is attributable to sitagliptin-induced ROS production, either due to the drug’s intrinsic effect alone or to impurities [24]. These results were critical for the design of our functional experiments, and in this context, we used a broad concentration range, from pharmacological to suprapharmacological levels, up to the maximal concentration (500 µg/mL), which was well below cytotoxic concentrations.

The primary findings of our study concerned the effects of sitagliptin on ROS production. Specifically, we assessed the kinetics of total ROS generation (luminol-enhanced chemiluminescence) and extracellular ROS release (isoluminol-enhanced chemiluminescence). Neutrophils were either left unstimulated or stimulated with PMA, fMLP, CaI, or OpsZym. These stimuli were chosen to probe distinct signaling pathways that differentially regulate the location and magnitude of ROS production [25].

Our data show that sitagliptin suppresses ROS production in human neutrophils under both basal (unstimulated) and activated conditions, reducing total ROS (luminol readout) and extracellular ROS release (isoluminol readout). In our culture system, even “unstimulated” neutrophils generated low but detectable ROS because isolation procedures and adherence during culture can prime/activate neutrophils [26,27]. Notably, inhibition of total ROS was generally evident at intermediate-to-higher concentrations (most often in the 62.5–500 range, depending on the experimental model), whereas extracellular ROS was attenuated even at lower concentrations, indicating a greater sensitivity of the released oxidant component to sitagliptin. Sitagliptin showed even greater ROS inhibition under HG conditions, a finding of great importance for neutrophil-mediated injury in diabetes [20].

Published data indicate that DPP-4 inhibitors can attenuate the oxidative burst in immune cells, although the magnitude of the response appears to depend on the compound structure and the cell type used in the experiments. In this context, linagliptin has been reported to markedly inhibit the oxidative burst and to reduce neutrophil adhesion to endothelial cells [28]. In contrast, a recent review demonstrates that linagliptin decreased ROS production in LPS-stimulated human neutrophils, whereas sitagliptin, vildagliptin, alogliptin, and saxagliptin showed little or no effect under the same conditions [8]. In monocytes, linagliptin decreased LPS-induced intracellular ROS production in human U937 cells [29]. In macrophage–lineage systems, anagliptin has been reported to reduce ROS production during macrophage differentiation by inhibiting NOX1/NOX2 [30], and a DPP-4 inhibitor analog (des-fluoro-sitagliptin) was reported to decrease macrophage ROS production in a model of atherosclerosis [31]. Gliptins were efficient suppressors of oxidative stress in experimental models of nonalcoholic cirrhosis and liver fibrosis [32]. In an in vitro study of polarized macrophages, sitagliptin reduced mitochondrial ROS [33].

In our system, the suppression of total ROS in neutrophil cultures by sitagliptin across all stimuli used is compatible with partial interference of this antidiabetic drug with NOX2 activation or assembly. This hypothesis is consistent with evidence that sitagliptin can reduce NADPH oxidase activity and blunt p47^phox translocation to the neutrophil membrane, a key step in oxidase assembly [34]. Sitagliptin attenuates arterial calcification by downregulating oxidative stress-induced receptor for advanced glycation end products in LDLR knockout mice [34]. Such an effect could dampen the NOX2 response on the plasma membrane elicited by PMA and fMLP. Mechanistically, PMA bypasses surface receptors and directly activates PKC, promoting NOX2 assembly by translocating cytosolic subunits to the plasma membrane and intracellular membrane complexes. This typically yields strong extracellular superoxide release in parallel with intracellular oxidant production [35,36,37]. In contrast, fMLP signals via formyl peptide receptors (GPCRs) and primarily promotes NOX2 assembly at the plasma membrane with rapid, predominantly extracellular superoxide release. In addition, it engages a PLC–IP_3_ pathway that triggers a transient cytosolic Ca^2+^ rise, which supports efficient NOX2 activation [38,39,40]. During OpsZym uptake, sitagliptin may attenuate the phagosomal NOX2 burst that is tightly coupled to phagocytosis. OpsZym is a particulate microbial stimulus that, once serum-opsonized, engages complement receptor 3 (CR3) and Fcγ receptors to drive phagocytosis-associated NOX2 assembly on the phagosomal membrane and triggers a vigorous respiratory burst. In parallel, phagosome–granule fusion delivers MPO that converts NOX2-derived H_2_O_2_ into HOCl. OpsZym-induced ROS products can also be detected extracellularly [25,41,42,43]. In addition, sitagliptin has been reported to reduce pro-oxidant signaling nodes, such as PKC activation, and to influence oxidative-stress mediators in diabetic tissues [44]. This finding could be relevant for decreased ROS production in our model of neutrophil culture under HG-like stress conditions.

Nothing is known about how sitagliptin modulates Ca signaling in neutrophils, a pathway relevant for CaI-induced ROS production. A23187 (Ca^2+^ ionophore) induces rapid Ca^2+^ influx and is generally linked to predominantly NOX-independent mitochondrial ROS generation. In addition, it can synergize with other stimuli via PKC to engage an NOX-2-dependent ROS pathway. Therefore, this pathway overlaps with PMA. Consequently, the ROS burst is usually biased toward intracellular sources with comparatively lower extracellular ROS [45,46,47]. However, some findings from other cellular models support sitagliptin’s interference with Ca mobilization by suppressing PLCγ2 phosphorylation [48]. Results from other models suggest that DPP4 inhibition could also protect mitochondrial Ca^2+^ homeostasis [49] and reduce mitochondrial ROS [33,50]. The relatively lower inhibitory effect of sitagliptin in PMA-stimulated neutrophils is likely due to the extremely high ROS production induced by PMA, which can reach up to 500-fold above unstimulated levels. Therefore, a maximal 50% reduction still represents a substantial inhibitory effect under these conditions.

A particularly relevant finding of our study is the ROS-scavenging activity of sitagliptin in cell-free systems, with only partial quenching of superoxide and H_2_O_2_ but a marked effect against HOCl. Notably, direct ROS-scavenging activity by gliptins has been far less extensively investigated. One study showed that teneligliptin directly scavenges hydroxyl radicals [51]. In neutrophils, HOCl is generated when myeloperoxidase (MPO) reacts with H_2_O_2_ to form the oxidized intermediate (Compound I), which then oxidizes chloride (Cl^−^) to HOCl. HOCl is a major and highly reactive downstream oxidant of the respiratory burst [52,53,54]. OpsZym-driven phagocytosis strongly activates the NOX2/H_2_O_2_/MPO cascade, leading to substantial formation of MPO-derived oxidants such as HOCl [52,53]. Therefore, if sitagliptin preferentially scavenges HOCl, it would be expected to produce a larger apparent extracellular ROS reduction in our experimental culture system under MPO-rich conditions. One limitation of this conclusion is that we used ascorbic acid as a positive control. Ascorbic acid itself can trigger oxidative stress in the presence of hydroxyl radical (°OH) and the iron traces that may be present in reagents or buffers. However, given the experimental conditions used (the use of very pure ≥ 99% ascorbic acid), this effect is expected to be minimal.

In summary, sitagliptin likely reduced extracellular ROS through a dual mechanism—direct scavenging of extracellular oxidants and suppression of ROS production, particularly by NOX2 at the plasma membrane. In addition, by lowering intracellular ROS generation, it would be expected to decrease H_2_O_2_ availability, which can diffuse out of the cytosol and contribute to the extracellular oxidant pool.

The second important finding from our study concerns the ability of sitagliptin to reduce NETosis. NETosis is a regulated neutrophil response in which cells release decondensed chromatin decorated with granule proteins to form NETs [55]. PMA is a prototypical, strong NETosis inducer, which drives NOX2-dependent ROS signaling that supports the canonical “suicidal” NET program [55,56]. In contrast, CaI induces a rapid Ca^2+^-driven NETosis pathway, which is often described as largely NOX2-independent and linked to mitochondrial ROS and peptidylarginine deiminase 4 (PAD4)-dependent chromatin decondensation. Its kinetics and morphology differ from those of PMA-induced NETosis [45,46]. fMLP, while a potent activator of neutrophil signaling and the oxidative burst, is generally considered a weak or insufficient NETosis inducer [38] and more often requires additional priming/co-stimulation to elicit NET release [57,58]. OpsZym is a particulate microbial stimulus that triggers phagocytosis and activates NOX2 during particle uptake. This generates abundant H_2_O_2_, which fuels MPO-dependent halogenation reactions, including HOCl, within the phagocytic compartment. Complement receptor signaling (e.g., via CR3) has also been implicated as an upstream pathway promoting NET formation in microbial settings. In addition, HOCl, generated by MPO released from azurophilic granules using H_2_O_2_ and chloride, can promote NET release under certain conditions [59,60].

Our finding that sitagliptin inhibits NETosis is broadly parallel to its inhibitory effects on ROS generation. These processes are tightly interconnected because ROS directly influences granule and nuclear envelope rupture [56]. This is especially relevant for spontaneous and PMA-, fMLP-, and OpsZym-induced NETosis. However, this comparison should be interpreted cautiously when analyzing CaI-induced NETosis, given differences in NET signaling pathways. In addition, neutrophils were incubated for a longer period in the NETosis assay than in the ROS assays, potentially increasing baseline DNA release over time. This likely explains the relatively higher proportion of NETosis observed in unstimulated samples (approximately 35% under NG conditions; 50% under HG conditions). This baseline is consistent with time-dependent spontaneous NET formation [61] and/or low-level activation during prolonged in vitro culture. It is also reasonable to add that reduced NETosis at the highest concentrations could partly reflect effects of sitagliptin on reduced neutrophil apoptosis, as we showed in our study, given the well-known mechanism of ROS-induced cell death [62].

Human neutrophils display highly dynamic surface receptors. After stimulation, including fMLP, they rapidly remodel adhesion molecules, chemoattractant and opsonin receptors, and granule-mobilization markers. These mechanisms include inside-out integrin activation, ectodomain shedding, receptor desensitization and/or internalization, and exocytosis of secretory vesicles and granules [63].

Our findings that fMLP upregulates CD11b/CD18 (Mac-1) and downregulates CD62L (L-selectin) define the classic reciprocal “early activation/priming” phenotype of neutrophils. CD62L supports neutrophil rolling and is rapidly shed after activation, reflecting the transition from rolling to firm adhesion [64]. Firm adhesion is mediated by the CD11b/CD18 integrin. In addition, this complement receptor 3 (CR3) is involved in neutrophil migration, ROS production, and phagocytosis [65]. CD11b/CD18 is typically up-regulated following mobilization from intracellular pools in response to fMLP stimulation [66].

Sitagliptin reduced the surface MFI of CD11b/CD18 in both unstimulated and fMLP-stimulated neutrophils. Because these changes occurred rapidly, they most likely reflect the inhibition of Mac-1 mobilization from intracellular pools to the plasma membrane and/or reduced inside-out integrin activation, rather than altered gene expression [67]. Functionally, lower surface Mac-1 indicates a reduced capacity for firm adhesion, crawling, and transendothelial recruitment. It can also blunt integrin-dependent amplification of neutrophil activation. This interpretation is consistent with our functional findings of reduced oxidative burst and reduced NET formation in neutrophil cultures preincubated with sitagliptin, since “primed” adhesive states, characterized by high Mac-1 availability and/or activation, are commonly linked to stronger downstream effector programs in neutrophils [68]. The somewhat different expression patterns of CD18 compared with CD11b in our study may be explained by the fact that CD18 is the common β2-integrin β chain shared by other α subunits (e.g., CD11a, CD11c, and CD11d) that are expressed on neutrophils [68].

In parallel, sitagliptin partially preserved CD62L (especially at lower concentrations), which is typically shed during activation. This suggests that sitagliptin limits the early transition from neutrophil rolling/tethering to firm integrin-mediated adhesion [69]. This supports the effect of sitagliptin in promoting an overall shift toward a less adhesive and less-primed neutrophil phenotype, consistent with our parallel functional findings of reduced oxidative burst and NET formation.

CD88 (C5aR1) is the fourth molecule in the recruitment/adhesion module investigated in this study. The finding that CD88 expression declines following fMLP stimulation is consistent with previous publications [65,70]. These results can be interpreted as desensitization and termination of C5aR1-mediated signaling via receptor internalization [71]. In addition, neutrophil serine proteases, such as neutrophil elastase and cathepsin, activated by fMLP, can cleave C5aR1, thereby reducing antibody recognition on the cell surface. Our findings that sitagliptin may preserve CD88 surface availability on both resting and fMLP-activated neutrophils suggest that the drug may maintain complement responsiveness while still dampening injurious effector outputs mediated by ROS and NETs. This is in agreement with the involvement of the C5a-C5aR1 axis in ROS/NET activation during inflammation [72,73].

CXCR1 (CD181) and CXCR2 (CD182) are the principal neutrophil receptors for ELR-positive (Glu–Leu–Arg–motif) CXC chemokines (including CXCL8/IL-8) [74]. Both IL-8 receptors mediate chemotaxis, but CD182 does not mediate neutrophil ROS production [75]. CXCR2 recognizes a broader range of ligands and internalizes markedly faster than CXCR1, with slower recycling [76]. These findings may explain our observation of the greater downregulation of CD182 following fMLP stimulation.

Sitagliptin generally increased CD181 expression on unstimulated neutrophils but decreased it in fMLP-stimulated cells. This may indicate that sitagliptin preserves baseline CXCR1 surface availability. However, when the activation signal is strong (fMLP), sitagliptin reduces signaling capacity by enhancing receptor desensitization and/or modulating receptor trafficking (increased internalization and slower return to the surface), thereby limiting CXCR1-driven amplification. The opposite results were observed with CD182. Sitagliptin decreased its expression under both unstimulated and fMLP-stimulated conditions. CD182 is more readily internalized from the membrane but is also mobilized more slowly from intracellular sources to the membrane than CD181. This may explain why the net effect of sitagliptin is a reduction in CXCR2, regardless of neutrophil activation status.

CD16 is a low-affinity Fcγ receptor on human neutrophils (FcγRIIIb) that binds IgG-containing immune complexes and opsonized particles, thereby supporting Fc-dependent neutrophil effector responses, such as phagocytosis and inflammatory responses. CD16 is rapidly down-modulated upon activation via ectodomain shedding (ADAM17-mediated cleavage) [77]. Our results in fMLP-treated cultures are consistent with these findings. We showed that sitagliptin reduced CD16 on resting neutrophils at higher concentrations, yet it increased CD16 on fMLP-stimulated cells across concentrations. These results suggest partial preservation of FcγRIIIb surface availability during activation. Possible mechanisms may include reduced receptor loss and/or altered receptor trafficking, rather than a uniform suppressive effect of sitagliptin.

CD66b is a granulocyte-restricted, GPI-anchored carcinoembryonic antigen-related cell adhesion molecule 8 (CEACAM8) that is stored in specific (secondary) granules [78]. It is rapidly up-regulated at the cell surface during degranulation, where it functions as an adhesion molecule [79]. Therefore, increased CD66b expression is recognized as a standard marker of neutrophil activation [78,79]. We confirmed this in our fMLP-stimulation model of neutrophils. Engagement of CD66b can also promote β2-integrin-dependent adhesion and trigger signaling pathways involved in neutrophil effector functions, such as IL-8 release [80]. We showed that sitagliptin, at the lowest concentration, decreased CD66b expression in both unstimulated and fMLP-stimulated neutrophils, suggesting that this antidiabetic drug may attenuate secondary granule degranulation.

CD63 is a tetraspanin enriched in azurophilic (primary) granule membranes. Increased surface CD63 is widely used as a flow cytometric indicator of primary granule exocytosis and plasma membrane fusion [78,81]. In our NG cultures, fMLP raised CD63 MFI, consistent with previous research showing that chemoattractant stimulation can promote primary granule mobilization and CD63 up-regulation. Similarly, CD89 (FcαRI), which supports IgA-opsonin recognition, also increased with fMLP activation, likely reflecting mobilization from intracellular pools within secretory vesicles and tertiary granules [70]. Our findings show that sitagliptin increases the expression of CD63 and CD89 on both unstimulated and fMLP-stimulated neutrophils. This is somewhat unexpected, given sitagliptin’s inhibitory effect on neutrophil function and other surface molecules. This may indicate altered mobilization of azurophilic granule-associated membranes and/or redistribution of intracellular pools, which can be at least partly separated from respiratory burst and NET formation. Similarly, higher CD89 surface levels should not be equated with improved FcαRI-dependent antimicrobial activity, as Fc receptor function depends not only on receptor levels but also on context-dependent inside-out signaling and ligand engagement [70]. Because both molecules are involved in phagocytosis and the subsequent killing of microorganisms [70,78,81], the functional significance of their increased expression cannot be determined without directly assessing sitagliptin’s effect on neutrophil phagocytic activity. Therefore, it remains unclear whether the observed up-regulation of these markers is associated with diminished or enhanced phagocytosis and bacterial killing. This is one of the limitations of the present study and an important issue to be addressed in future investigations.

CD10 (neprilysin) is often used as a marker of neutrophil maturity [82]. Adult neutrophils increase CD10 MFI after fMLP stimulation [83], a pattern consistent with our findings. An additional increase in CD10 on both resting and fMLP-stimulated neutrophils after sitagliptin treatment can be attributed to increased mobilization from intracellular compartments, including secretory vesicles [83,84]. The rise in CD10 expression may also indicate changes in maturation status and regulatory peptide processing, rather than just activation. This finding is consistent with the increased immunoregulatory role of CD10+ neutrophils in inhibiting T cell proliferation, in contrast to CD10- neutrophils, which have pro-inflammatory properties [82]. It seems that sitagliptin’s effect on CD10 expression is similar to its effects on the other two markers (CD63 and CD89), and the possible coordinated action of all three molecules may contribute to the increased immunosuppressive mechanisms of this antidiabetic drug, which is intriguing for further studies.

An increase in PD-L1, a known checkpoint with immunoregulatory properties, on both resting and fMLP-stimulated neutrophils after sitagliptin treatment is consistent with the drug’s previously reported immunomodulatory properties [22]. Some reports showed that increased PD-L1 expression on neutrophils correlates with better antimicrobial properties [85]. However, the role of PD-L1 in our experimental model is likely less important given its lower expression.

Our findings show that neutrophil phenotypic characteristics differ in HG medium; however, the overall pattern of marker modulation after fMLP stimulation is generally similar to that observed in NG medium. Still, some differences stand out, including lower expression of CD11b, CD88, and CD181, and higher expression of CD63, CD16b, and PD-L1. These findings may be linked to the well-documented priming of neutrophils by higher glucose concentrations, which alters their activation status [20].

As a rule, sitagliptin exerted weaker immunomodulatory effects on neutrophil phenotypes in HG medium. However, the available literature on this topic is very limited. In one study, no changes in the phenotypes of CD11b and CD66b on neutrophils were observed in HG medium, although their expression increased in patients with diabetes [86]. Our finding of no modulation of CD181 and CD182 expression may relate to results from a diabetes-with-sepsis model, in which internalization of the chemokine receptor CXCR2 is reported, leading to a consequent reduction in neutrophil chemotaxis.

Because membrane CD26/DPP-4 was not detected on neutrophils in our system, a direct DPP-4-dependent surface mechanism seems less likely. An alternative explanation is that the observed effects may involve intracellular DPP-8/9, although this remains speculative and would need direct experimental confirmation. Future studies should therefore evaluate DPP-8/9 expression and activity in neutrophils and compare the effects of sitagliptin with available DPP-8/9-selective pharmacological tools, such as 1G244, and, if feasible, more DPP8-biased analogs [87]. Additionally, DPP4 inhibitors have been reported to reduce oxidative/inflammatory signaling by affecting the Nrf2/NF-κB/NADPH oxidase-related pathways. As discussed, these pathways are significantly involved in many neutrophil functions, including ROS production, NETosis, chemotaxis, degranulation, and others [88,89]. At the same time, pathway-focused analyses should be conducted to determine if sitagliptin influences NF-κB and Nrf2 signaling, for example, by examining p65 and Nrf2 nuclear translocation, IκBα degradation, and downstream anti-oxidant targets. Assessing autophagy-related markers would also be relevant, given the known interplay between ROS production and autophagy and the reported connection of sitagliptin with the p62–Keap1–Nrf2 signaling axis [88].

A question was raised as to whether the in vitro concentrations used were clinically relevant, given that sitagliptin is typically administered at a daily dose of 100 mg for the treatment of T2DM. However, when sitagliptin is considered for immunomodulatory purposes, such as in clinical allograft transplantation studies to prevent acute graft-versus-host disease, considerably higher doses (600 mg twice daily) have been shown to provide effective immunosuppression with only minimal adverse effects [90]. These findings support the relevance of higher sitagliptin concentrations for investigations of immunomodulatory activity.

In conclusion, our study demonstrated a marked inhibitory effect of sitagliptin on ROS production and NETosis, along with a complex modulatory effect on the expression of membrane markers on both resting and stimulated human peripheral blood neutrophils cultured under NG and HG conditions. We also showed the significant ROS-scavenging effect of sitagliptin. To our knowledge, these findings have not been reported previously and, overall, support the anti-inflammatory and immunomodulatory properties of this antidiabetic drug. This is significant given that T2DM very often induces a systemic inflammatory response. In addition, the obtained results suggest potential additional indications for the use of sitagliptin as an immunomodulatory agent, although further studies are needed to confirm this. The study raises several additional questions regarding the mechanisms of sitagliptin’s action, which represent a key limitation of the present work and should be addressed in future research.

## 4. Materials and Methods

### 4.1. Ethics Statement

Research was conducted at the Center for Biomedical Research, University of East Sarajevo, Faculty of Medicine, Foča. Blood was drawn from healthy volunteers by venipuncture into K2EDTA tubes as an anticoagulant. All participants signed the Informed Consent Form in accordance with the Declaration of Helsinki. Approval for the study was granted by the Ethics Committee of the Faculty of Medicine in Foča (number: 01-2-33/05.06.2023).

### 4.2. Isolation of Peripheral Blood Neutrophils

Neutrophil granulocytes (neutrophils) from peripheral blood were isolated from healthy donors (n = 3 for functional assays; n = 4 for flow cytometric assays). The donors were of both sexes, aged 21–25 years, who did not take antibiotics or immunomodulatory therapy within the last four months. All blood samples were used immediately after collection and separated by sedimentation in a 3% (*w*/*v*) dextran solution (Sigma-Aldrich, Steinheim, Germany). Following sedimentation, leukocyte-rich plasma was collected and layered onto a Lymphoprep gradient (1.077 g/mL; PAA Laboratories, Vienna, Austria). Neutrophils were separated by centrifugation (2200 rpm for 20 min at room temperature) and residual erythrocytes were lysed with a lysing solution (NH_4_Cl, KHCO_3_, and Na_2_EDTA; pH 7.4) for 2 min. Neutrophils were resuspended in Hank’s balanced salt solution without Ca^2+^ and Mg^2+^ (HBSS^−^; Sigma-Aldrich) and used for further experimental purposes. Cell viability was assessed by staining with a 0.2% (*w*/*v*) trypan blue solution (Sigma-Aldrich). Cell counts were determined using a Neubauer counting chamber and a light microscope. Cytospins stained with May-Grunwald Giemsa revealed that the isolated fraction contained more than 95% neutrophils.

### 4.3. Cytotoxicity Assays

The cytotoxicity of sitagliptin phosphate monohydrate (Sigma-Aldrich) was assessed using a commercial apoptosis/necrosis kit. We used the Annexin V/Propidium Iodide (PI) staining kit (BioLegend, London, UK) according to the manufacturer’s instructions to detect necrosis and apoptosis. Neutrophils (3 × 10^5^ cells/100 µL) were incubated in polypropylene 2 mL tubes (Eppendorf, Hamburg, Germany) for 30 min at 37 °C and 5% CO_2_ in normal glucose (NG, 5.5 mM) (glucose stock, 278 mM, resuspended in HBSS^+^ medium (with Ca^2+^ and Mg^2+^ enriched with 1% heat-inactivated human serum and 10 mM HEPES) to a final concentration of 5.5 mM) and high glucose (HG, 22 mM) (glucose stock, 278 mM, resuspended in HBSS^+^ medium to a final concentration of 22 mM) in a total volume of 150 µL, and incubated for 2 h at 37 °C and 5% CO_2_.

After incubation, the cells were treated with various concentrations of sitagliptin (Sigma-Aldrich). Sitagliptin was dissolved in sterile distilled water to prepare a 50 mg/mL stock solution. Final concentrations of 3.6, 7.5, 15, 30, 62.5, 125, 250, 500, 1000, and 2000 µg/mL (corresponding concentrations in µM: 6.88, 14.33, 28.66, 57.33, 119.43, 238.86, 477.72, 955.44, 1910.88, and 3821.75) were prepared in a final volume of 50 µL, and cells were incubated for 6 h at 37 °C and 5% CO_2_. Alternatively, cells were stimulated with fMLP and incubated for an additional 16 h. Following incubation, neutrophils were washed, and, according to the manufacturer’s instructions, binding buffer from the commercial kit and fluorescent dyes were added to the cell pellet. After a 20 min incubation in the dark, the samples were washed in binding buffer and analyzed using a flow cytometer (Attune, Thermo Fisher Scientific, Waltham, MA, USA).

The following cell classifications were used: necrotic cells positive only for PI (Annexin V FITC^−^/PI^+^), late apoptotic cells positive for both Annexin V and propidium iodide (Annexin V FITC^+^/PI^+^), and early apoptotic cells positive for Annexin V–fluorescein isothiocyanate (Annexin V FITC^+^/PI^−^). Data analysis was performed using FlowJo X software (v11.1.0, BD Biosciences, Franklin Lakes, NJ, USA), and the results are presented as percentages.

### 4.4. The Production of Reactive Oxygen Species (ROS)

We used a luminol- and isoluminol-based chemiluminescence assay to detect total and extracellular ROS. Following the previously described protocol, neutrophils were isolated from the peripheral blood of healthy volunteers and resuspended in HBSS^+^ medium. The cells were then plated in 96-well flat-bottomed white opaque plates (Corning, New York, NY, USA) at a density of 2.5 × 10^5^ cells/50 µL per well. After incubation (30 min at 37 °C and 5% CO_2_), the cells were exposed to normal glucose (NG, 5.5 mM) or high glucose (HG, 22 mM) in 150 µL and incubated for 2 h at 37 °C and 5% CO_2_.

After incubation, the cells were treated with varying doses of sitagliptin at final concentrations of 7.5, 15, 30, 62.5, 125, 250, and 500 µg/mL in 50 µL and incubated for 1 h at 37 °C and 5% CO_2_. Following incubation, 25 µL of luminol (50 µM; Serva, Munich, Germany) or isoluminol (50 µM; Serva) combined with horseradish peroxidase (HRP, 4 U/mL) (Serva) in HBSS^+^ medium was added to each well, and the mixture was incubated for 15 min. Cells stimulated with PMA/fMLP/CaI/OpsZym in the absence of sitagliptin served as positive controls for ROS production by activated neutrophils.

After 15 min, the cells were stimulated with the following stimuli, resuspended in HBSS^+^ medium to a final volume of 25 µL, and incubated at 37 °C and 5% CO_2_: phorbol myristate acetate (PMA, 50 nM; Sigma-Aldrich), calcium ionophore A23187 (CaI, 1 µM; Sigma-Aldrich), N-formyl-methionyl-leucyl-phenylalanine (fMLP, 1 µM; Sigma-Aldrich), and opsonized zymosan (OpZym, 10 µg; Sigma-Aldrich). We did not use fMLP-primed conditions, such as preincubation with cytochalasin B, to better mimic the physiological action of fMLP.

The intensity of light emitted was measured immediately after stimulation using a chemiluminescence plate reader (Synergy HTX, BioTek Instruments, Santa Clara, CA, USA). The emitted light intensity was proportional to the formation of ROS. ROS production was measured every 2 min for 3 h at 37 °C. Results are presented as the area under the curve (AUC).

Intracellular ROS production was further assessed using DHR 123 (Sigma-Aldrich) staining. Neutrophil granulocytes (4 × 10^5^ cells in 100 µL) were incubated in HBSS medium in 2 mL polypropylene tubes for 30 min at 37 °C with 5% CO_2_. After this initial incubation, sitagliptin (62.5, 125, 250, and 500 µg/mL) was added in a volume of 100 µL, and cells continued to incubate for an additional hour. Some samples remained unstimulated, while others were stimulated with fMLP (1 µM). DHR (1 µM) and fMLP (1 µM) were added simultaneously in 25 µL volumes each, and the mixture was incubated for 15 min at 37 °C with 5% CO_2_. After incubation, cells were placed on ice in the dark for 20 min, then washed with cold PBS. Finally, intracellular ROS levels were measured by flow cytometry, with results expressed as mean fluorescence intensity (MFI). Data analysis was performed using FlowJo X software.

### 4.5. ROS-Scavenging Assays

#### 4.5.1. Hydrogen Peroxide (H_2_O_2_) Scavenging Assay

The H_2_O_2_ scavenging activity was determined by measuring H_2_O_2_-induced oxidation of lucigenin using a previously established chemiluminescence method with minor modifications. In a white 96-well plate, sitagliptin (1.62, 3.75, 7.5, 15, 30, 62.5, 125, 250, 500, and 1000 µg/mL) was added at 100 µL per well, and 100 µL of ascorbic acid (L-ascorbic acid, 500 µM; stock solution, 568 mM) of high purity (≥99%, MFCD00064328, Sigma-Aldrich), served as a positive control. In each well, 100 µL of 3% (*v*/*v*) hydrogen peroxide was added, followed by 50 µM luminol (50 mM stock solution). The experiments were performed at 37 °C. Chemiluminescence signals were measured using a microplate reader immediately after the plate was inserted. Each study consisted of three independent experiments performed in triplicate.

#### 4.5.2. Hypochlorous Acid (HOCl) Scavenging Assay

HOCl was detected using a previously established fluorescence-based method that relies on HOCl-induced oxidation of dihydrorhodamine (DHR; Sigma-Aldrich) to rhodamine 123, adapted for use in a microplate reader. In a black 96-well plate, 100 µL of sitagliptin (1.62, 3.75, 7.5, 15, 30, 62.5, 125, 250, 500, and 1000 µg/mL) and 100 µL of L-ascorbic acid (positive control) were added to each well. HOCl was prepared immediately before use by adjusting the pH of a 1% NaOCl solution to 6.2 with dropwise addition of 10% H_2_SO_4_. DHR (5 µM) was resuspended in HBSS^+^ medium and added to each well. Fluorescence measurements were performed at 37 °C using an excitation wavelength of 485 ± 20 nm and an emission wavelength of 528 ± 20 nm. Fluorescence signals were recorded immediately after plate insertion. Results are presented as the percentage inhibition of DHR oxidation induced by HOCl. Each study consisted of three independent experiments performed in triplicate.

#### 4.5.3. Superoxide Radical (O_2_•^−^) Scavenging Assay

Superoxide radicals were generated using the NADH/phenazine methosulfate (PMS) system (Sigma-Aldrich, Steinheim, Germany), and O_2_•^−^ scavenging activity was measured spectrophotometrically in a microplate reader by monitoring the effect of sitagliptin on O_2_•^−^-induced reduction in nitroblue tetrazolium (NBT; Sigma-Aldrich) at 560 nm for 2 min.

In a 96-well plate, 100 µL of sitagliptin (1.62, 3.75, 7.5, 15, 30, 62.5, 125, 250, 500, and 1000 µg/mL) and 100 µL of L-ascorbic acid (positive control) were added. Sample wells contained the following reactants at final concentrations (final volume 250 µL): NADH (166 µM), NBT (43 µM), and PMS (2.7 µM). The experiment was performed at room temperature. Results are expressed as the percentage inhibition of NBT reduction to formazan. Each study consisted of three independent experiments performed in triplicate.

### 4.6. Formation of Neutrophil Extracellular Traps (NETs)

In a 96-well black flat-bottomed plate (Corning), neutrophil granulocytes (1.5 × 10^5^ cells/50 µL) were resuspended in HBSS^+^ and incubated for 30 min at 37 °C and 5% CO_2_. Following incubation, the cells were cultured for 2 h in 150 µL of HBSS^+^ medium at either a normal-glucose concentration (NG, 5.5 mM) or a high-glucose concentration (HG, 22 mM). Following incubation, sitagliptin (50 µL) at different final concentrations (7.5, 15, 30, 62.5, 125, 250, and 500 µg/mL) was added, and the cells were incubated for an additional 1 h at 37 °C and 5% CO_2_. Following sitagliptin treatment, neutrophils were stimulated with CaI (1 µM), PMA (50 nM), fMLP (1 µM), or OpZym (10 µg) in a final volume of 25 µL and incubated at 37 °C and 5% CO_2_. In parallel, the same cultures were left unstimulated under identical incubation conditions. Certain wells were treated with 1% Triton X-100 (Sigma-Aldrich) to determine the total DNA content. After a 4 h incubation period, the fluorescent dye Sytox Green (Invitrogen/Thermo Fisher Scientific, Waltham, MA, USA) (5 µM) in a final volume of 25 µL was added, and fluorescence was measured using a fluorimeter plate reader. The degree of NETosis is directly proportional to fluorescence intensity.

### 4.7. Flow Cytometry-Based Phenotypic Analysis of Neutrophils

A panel of monoclonal antibodies and flow cytometry was used to determine the phenotypic properties of neutrophil granulocytes, stimulated with fMLP or unstimulated, under different glucose conditions. We measured the expression of several neutrophil markers involved in neutrophil recruitment and adhesion, including CD62L-PE (L-selectin; 1:100), CD11b-PerCP-Cy5 (β2 integrin; 1:100), CD18-Alexa Fluor 700 (1:100), and CD88-FITC (complement component 5a receptor, C5aR1; 1:100). Four markers involved in neutrophil chemotaxis, opsonization, and degranulation were analyzed: CD181-FITC (high-affinity IL-8 receptor, CXCR1; 1:100), CD182-FITC (high-affinity IL-8 receptor, CXCR2; 1:100), CD16-APC (Fc fragment of IgG receptor, FcγRIII; 1:400), and CD66b-APC (marker of specific granule degranulation; 1:100). In addition, four markers involved in neutrophil effector readiness and regulatory phenotype were analyzed: CD63-Alexa Fluor 700 (1:100), CD89-PerCP-Cy5 (Fc fragment of IgA receptor; 1:100), CD10-APC (1:100), and CD274-PE (PD-L1; 1:100). All antibodies were purchased from BioLegend (BioLegend, London, UK).

Neutrophil granulocytes (5 × 10^5^ cells in 300 µL) were cultured in HBSS^+^ medium in 2 mL polypropylene tubes for 2 h at 37 °C and 5% CO_2_ under normal-glucose (NG, 5.5 mM) or high-glucose (HG, 22 mM) conditions. Following incubation, sitagliptin (62.5, 125, 250, and 500 µg/mL) was added, and cells were incubated for an additional 1 h. Some cultures remained unstimulated, while others were stimulated with fMLP (1 µM) for an additional 1 h. After incubation, cells were washed in cold phosphate-buffered saline (PBS) containing 2% fetal calf serum (FCS) and 0.01% sodium azide (NaN_3_). Blocking antibodies against the Fc fragment were added at a dilution of 1:50 and incubated at 4 °C for 25 min. Subsequently, monoclonal antibodies were added at the indicated dilutions according to the manufacturer’s instructions and incubated at 4 °C for 30 min. Cells were then analyzed by flow cytometry.

Results were presented as mean fluorescence intensity (MFI), which reflects the surface expression density of each marker. Neutrophils were gated by excluding doublets and dead cells based on forward- and side-scatter characteristics. Before each experiment, single-stain controls were used to correct for signal overlap between fluorescence channels. Data analysis was performed using FlowJo X software.

### 4.8. Statistics

All statistical analyses were performed using GraphPad Prism 9.1 (GraphPad, La Jolla, CA, USA). For quantitative variables, the mean and standard deviation were used to describe the distribution. Normality was confirmed using the Kolmogorov–Smirnov test and the D’Agostino–Wilkinson–Lillie *p*-value. Group differences were assessed using Student’ *t*-test for independent groups (normally distributed variables) or the Mann–Whitney U test for non-normally distributed variables. For normally distributed groups, we used a one-way ANOVA with Dunnett’s multiple-comparison test. *p*-values < 0.05 were considered statistically significant. Results are shown as representative data or mean ± SD values from three independent trials.

## Figures and Tables

**Table 1:** Effect of sitagliptin on total apoptosis in unstimulated and fMLP-stimulated neutrophils under normal- and high-glucose conditions.

Sitagliptin (µg/mL)	Unstimulated—Total Apoptosis (%) (NG)	fMLP—Total Apoptosis (%) (NG)	Unstimulated—Total Apoptosis (%) (HG)	fMLP—Total Apoptosis (%) (HG)
0	55.0 ± 6.8	63.7 ± 6.5	56.0 ± 6.5	63.5 ± 5.2
3.7	50.7 ± 7.5	62.8 ± 7.3	58.7 ± 6.7	58.5 ± 6.4
7.5	57.5 ± 6.1	63.8 ± 5.2	55.9 ± 6.0	59.0 ± 6.3
15	49.8 ± 5.3	65.8 ± 4.8	48.0 ± 6.7	63.8 ± 4.7
30	61.4 ± 7.0	60.7 ± 7.4	58.8 ± 4.7	48.4 ± 9.5
62.5	58.3 ± 4.0	61.3 ± 6.7	51.9 ± 8.8	56.4 ± 5.7
125	54.1 ± 7.7	63.2 ± 6.6	51.3 ± 8.6	51.5 ± 6.5
250	50.3 ± 7.4	53.7 ± 5.5	49.6 ± 6.5	48.5 ± 7.5
500	48.1 ± 4.9 *	55.9 ± 4.1 *	53.4 ± 5.2	48.8 ± 4.9 *
1000	48.1 ± 5.1 *	46.6 ± 5.7 **	36.6 ± 5.8 *	38.1 ± 5.8 ***
2000	70.4 ± 5.3 **	76.2 ± 4.4 *	77.2 ± 5.0 ***	83.0 ± 4.8 ***

Data are presented as mean ± SD (*n* = 3 different donors). Statistical significance relative to the corresponding control (0 µg/mL) is indicated as follows: * *p* < 0.05, ** *p* < 0.01, and *** *p* < 0.005. Total apoptosis was calculated as the sum of Annexin V^+^/PI^−^ (early apoptotic) and Annexin V^+^/PI^+^ (late apoptotic/secondary necrotic) populations. NG: normal-glucose; HG: high-glucose; fMLP: formyl-methionyl-leucyl-phenylalanine.

## Data Availability

The original contributions presented in this study are included in the article/Supplementary Materials. Further inquiries can be directed to the corresponding author.

## Supplementary Materials

The following supporting information can be downloaded at https://www.mdpi.com/article/10.3390/molecules31081257/s1; Figure S1: Effect of varying sitagliptin concentrations on apoptosis and necrosis of human peripheral blood neutrophils. Neutrophils were incubated in normal (NG, 5.5 mM) or high-glucose (HG, 22 mM) media and treated with sitagliptin at doubling concentrations from 3.7 to 1000 µg/mL. Cells were either non-stimulated (NS) or stimulated with fMLP for 16 h, as described in Section 4. Cytotoxicity was assessed using an apoptosis/necrosis assay. Representative plots of apoptosis and necrosis from one experiment; Figure S2: The effect of sitagliptin on human neutrophil ROS production. Cells were grown in HBSS^+^ medium and then treated with sitagliptin at concentrations from 62.5 to 500 µg/mL for 1 h. After incubation, some cultures remained non-stimulated (NS), while others were stimulated with fMLP (1 µM). DHR (1 µM), a fluorescent dye, was added simultaneously. After 20 min of incubation at 37 °C, cells were placed on ice for 15 min and then washed with cold PBS. Fluorescence intensity was measured by flow cytometry and reported as mean fluorescence intensity (MFI). Representative histograms from one experiment are shown; Figure S3: Effect of sitagliptin on the surface expression of CD11b, CD18, CD62L, and CD88. Human neutrophils were cultured in HBSS+ medium under normal-glucose (NG, 5.5 mM) or high-glucose (HG, 22 mM) conditions for 2 h and then treated with sitagliptin (62.5, 125, 250, and 500 µg/mL) for 60 min. Cells were left unstimulated (NS) or stimulated with fMLP. Surface expression of CD11b, CD18, CD62L, and CD88 was analyzed by flow cytometry and expressed as mean fluorescence intensity (MFI). Representative histograms for the highest sitagliptin concentration (500 µg/mL) are shown; Figure S4: Effect of sitagliptin on the surface expression of CD181, CD182, CD16, and CD66b. Human neutrophils were cultured in HBSS^+^ medium under normal-glucose (NG, 5.5 mM) or high-glucose (HG, 22 mM) conditions for 2 h and then treated with sitagliptin (62.5, 125, 250, and 500 µg/mL) for 60 min. Cells were left unstimulated (NS) or stimulated with fMLP. Surface expression of CD181, CD182, CD16, and CD66b was analyzed by flow cytometry and expressed as mean fluorescence intensity (MFI). Representative histograms for the highest sitagliptin concentration (500 µg/mL) are shown; Figure S5: Effect of sitagliptin on the surface expression of CD63, CD89, CD10, and PD-L1. Human neutrophils were cultured in HBSS^+^ medium under normal-glucose (NG, 5.5 mM) or high-glucose (HG, 22 mM) conditions for 2 h and subsequently treated with sitagliptin (62.5, 125, 250, and 500 µg/mL) for 60 min. Cells were left unstimulated (NS) or stimulated with fMLP. Surface expression of CD63, CD89, CD10, and PD-L1 was analyzed by flow cytometry and expressed as mean fluorescence intensity (MFI). Representative histograms for the highest sitagliptin concentration (500 µg/mL) are shown.
