# Supplementary material for: Sitagliptin Modulates Functional and Phenotypic Properties of Human Neutrophils Under Normal- and High-Glucose Conditions In Vitro
Source: Molecules. 2026 Apr 10;31(8):1257. doi: 10.3390/molecules31081257 (PMC13118998; doi:10.3390/molecules31081257)
Supplement: Supplementary file 1 [file molecules-31-01257-s001.zip › molecules-4198193-supplementary.pdf]

Supplementary Materials

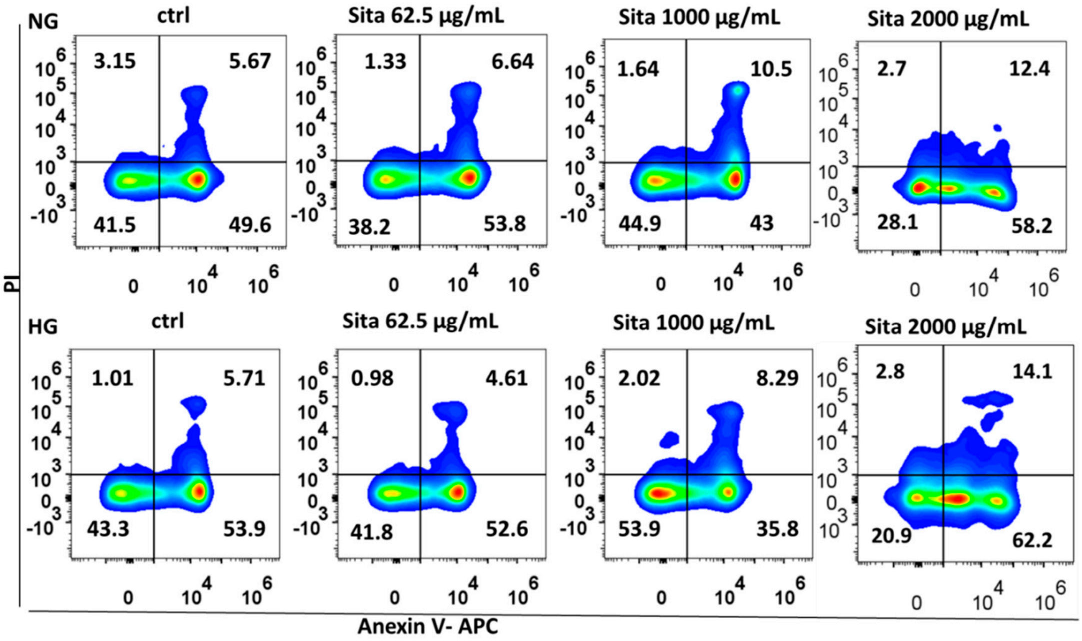

Supplementary Figure S1. Effect of varying sitagliptin concentrations on apoptosis and necrosis of human peripheral blood neutrophils. Neutrophils were incubated in normal (NG, 5.5 mM) or high-glucose (HG, 22 mM) media and treated with sitagliptin at doubling concentrations from 3.7 to 1000 µg/mL. Cells were either non-stimulated (NS) or stimulated with fMLP for 16 h, as described in the Materials and Methods section. Cytotoxicity was assessed using an apoptosis/necrosis assay. Representative plots of apoptosis and necrosis from one experiment.

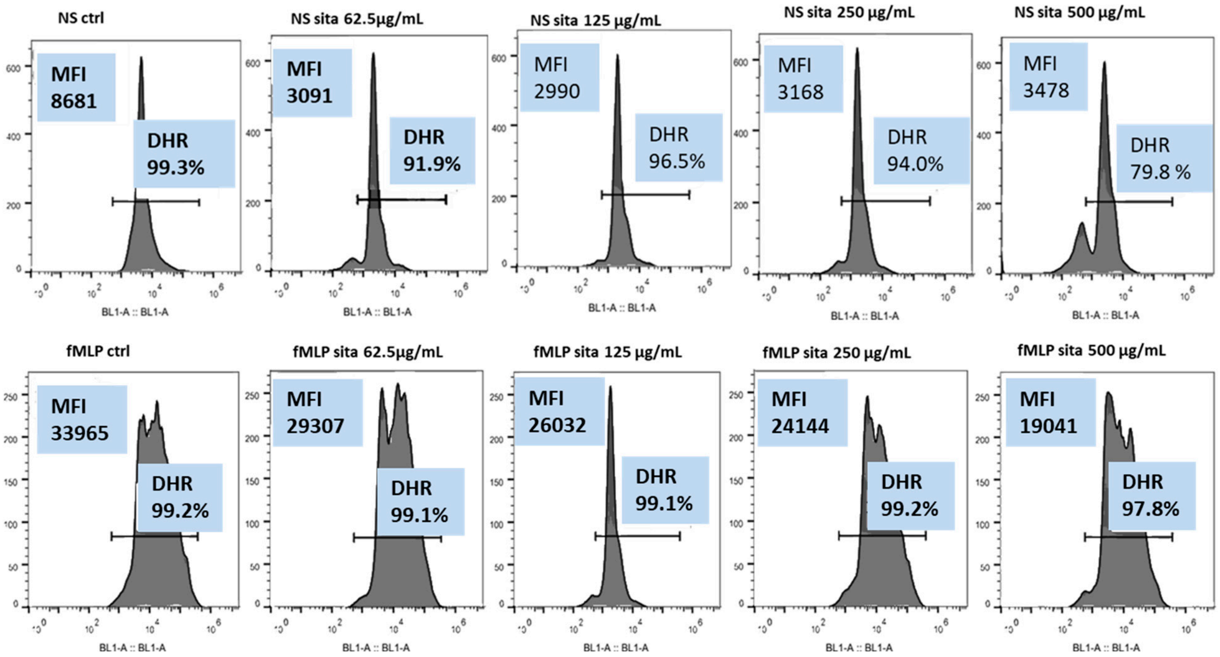

Supplementary Figure S2. The effect of sitagliptin on human neutrophil ROS production. Cells were grown in HBSS$^+$ medium and then treated with sitagliptin at concentrations from 62.5 to 500 µg/mL for 1 hour. After incubation, some cultures remained non-stimulated (NS), while others were stimulated with fMLP (1 µM). DHR (1 µM), a fluorescent dye, was added at the same time. After 20 minutes of incubation at 37 °C, cells were placed on ice for 15 minutes and then washed with cold PBS. Fluorescence intensity was measured by flow cytometry and reported as mean fluorescence intensity (MFI). Representative histograms from one experiment are shown.

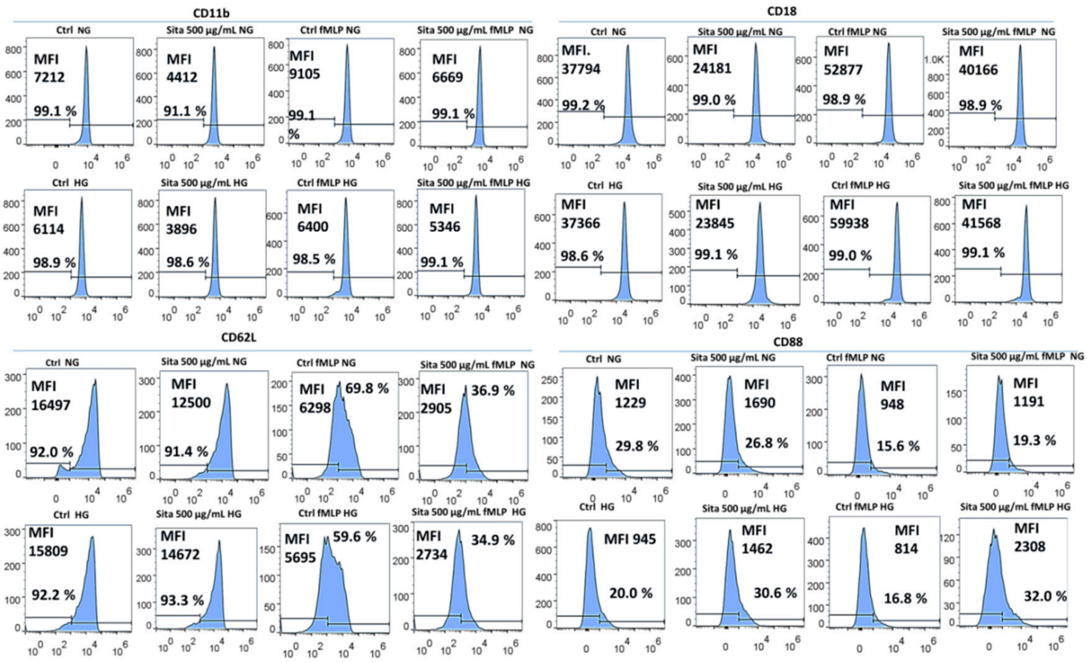

Supplementary Figure S**3**. Effect of sitagliptin on the surface expression of CD11b, CD18, CD62L, and CD88. Human neutrophils were cultured in HBSS$^+$ medium under normal glucose (NG, 5.5 mM) or high glucose (HG, 22 mM) conditions for 2 h and then treated with sitagliptin (62.5, 125, 250, and 500 μg/mL) for 60 min. Cells were left unstimulated (NS) or stimulated with fMLP. Surface expression of CD11b, CD18, CD62L, and CD88 was analyzed by flow cytometry and expressed as mean fluorescence intensity (MFI). Representative histograms for the highest sitagliptin concentration (500 μg/mL) are shown.

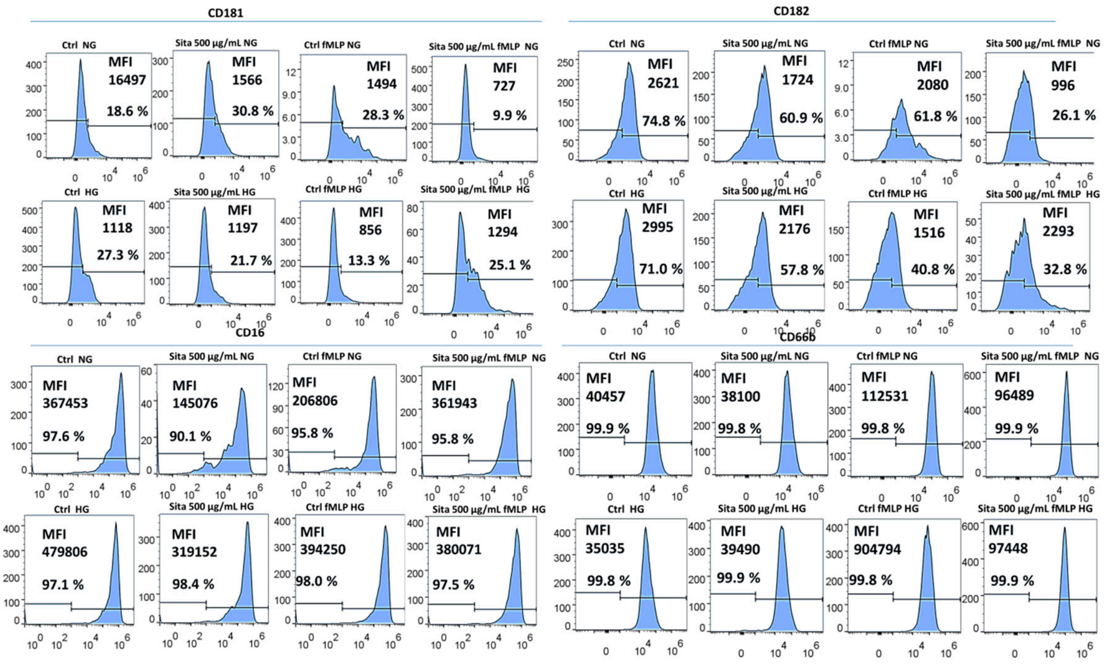

Supplementary Figure S4. Effect of sitagliptin on the surface expression of CD181, CD182, CD16, and CD66b. Human neutrophils were cultured in $HBSS^+$ medium under normal glucose (NG, 5.5 mM) or high glucose (HG, 22 mM) conditions for 2 h and then treated with sitagliptin (62.5, 125, 250, and 500 µg/mL) for 60 min. Cells were left unstimulated (NS) or stimulated with fMLP. Surface expression of CD181, CD182, CD16, and CD66b was analyzed by flow cytometry and expressed as mean fluorescence intensity (MFI). Representative histograms for the highest sitagliptin concentration (500 µg/mL) are shown.

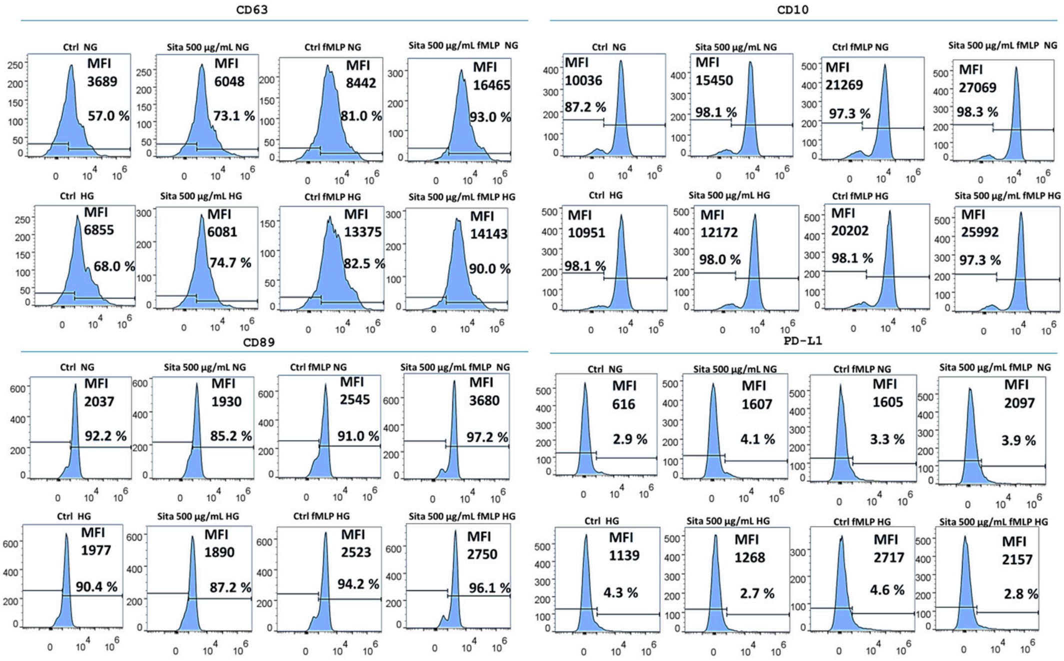

Supplementary Figure S5. Effect of sitagliptin on the surface expression of CD63, CD89, CD10, and PD-L1. Human neutrophils were cultured in HBSS+ medium under normal glucose (NG, 5.5 mM) or high glucose (HG, 22 mM) conditions for 2 h and subsequently treated with sitagliptin (62.5, 125, 250, and 500 µg/mL) for 60 min. Cells were left unstimulated (NS) or stimulated with fMLP. Surface expression of CD63, CD89, CD10, and PD-L1 was analyzed by flow cytometry and expressed as mean fluorescence intensity (MFI). Representative histograms for the highest sitagliptin concentration (500 µg/mL) are shown.
